# Repression of ZNFX1 by LncRNA ZFAS1 mediates tobacco-induced pulmonary carcinogenesis

**DOI:** 10.1186/s11658-025-00705-x

**Published:** 2025-04-10

**Authors:** Sichuan Xi, Jigui Shan, Xinwei Wu, Haitao Wang, Mary R. Zhang, Shakirat Oyetunji, Hong Xu, Zuoxiang Xiao, Tuana Tolunay, Shamus R. Carr, Chuong D. Hoang, David S. Schrump

**Affiliations:** 1 Thoracic Epigenetics Section, Thoracic Surgery Branch, Center for Cancer Research, National Cancer Institute, Building 10; 4-3942, 10 Center Drive, Bethesda, MD 20892 USA; 2 Advanced Biomedical Computational Science, Frederick National Laboratory for Cancer Research, Frederick, MD 21702 USA; 3 Laboratory of Cancer Prevention, National Cancer Institute, Frederick, MD 21702 USA; 4 Cancer and Inflammation Program, Center for Cancer Research, National Cancer Institute, Frederick, MD 21702 USA

**Keywords:** Lung cancer, Epigenetics, Noncoding RNA, *ZNFX1*, *ZFAS1*, Cigarette smoke, EZH2, BMI1, SUZ12, DNMT, SP1, NFĸB

## Abstract

**Background:**

Despite exhaustive research efforts, integrated genetic and epigenetic mechanisms contributing to tobacco-induced initiation and progression of lung cancers have yet to be fully elucidated. In particular, limited information is available regarding dysregulation of noncoding RNAs during pulmonary carcinogenesis.

**Methods:**

We examined correlations and interactions of long noncoding (lnc) RNAs and protein-coding genes in normal respiratory epithelial cells (NREC) and pulmonary tumor cells following exposure to cigarette smoke condensate (CSC) using gene expression arrays, qRT-PCR, western blot, growth assays, transwell assays, and murine xenograft models, as well as methylated DNA immunoprecipitation, RNA cross-link immunoprecipitation, and quantitative chromatin immunoprecipitation techniques with bioinformatics analyses.

**Results:**

Among diverse alterations of lncRNA and coding gene expression profiles in NREC exposed to CSC, we observed upregulation of lncRNA ZFAS1 and repression of an adjacent protein-coding gene, *ZNFX1*, and confirmed these findings in primary lung cancers. Phenotypic experiments indicated that *ZFAS1* is an oncogene, whereas *ZNFX1* functions as a tumor suppressor in lung cancer cells. Mechanistically, CSC induces *ZFAS1* expression via SP1 and NFĸB-associated activation of an enhancer linked to *ZFAS1*. Subsequently, ZFAS1 interacts with DNA methyltransferases and polycomb group proteins to silence *ZNFX1*. Mithramycin and methysticin repress *ZFAS1* and upregulate *ZNFX1* in lung cancer cells in vitro and in vivo.

**Conclusion:**

These studies reveal a novel feedforward lncRNA circuit contributing to pulmonary carcinogenesis and suggest that pharmacologic targeting of SP1 and/or NFĸB may be useful strategies for restoring *ZNFX1* expression for lung tumor therapy.

**Supplementary Information:**

The online version contains supplementary material available at 10.1186/s11658-025-00705-x.

## Introduction

Lung cancer continues to be the leading risk factor of global cancer-related mortality, claiming over 1.8 million lives annually, of which 160,000 deaths occur each year in the USA [1, 2]. These cancers are generally categorized into two major subtypes based on histologic, molecular, and clinical characteristics. Non-small cell lung cancers (NSCLC) including adenocarcinomas, squamous cell carcinomas, and large cell undifferentiated carcinomas constitute roughly 85% of pulmonary malignancies, whereas small cell lung cancers (SCLCs) with varying neuroendocrine features account for the remaining 15% [3, 4]. Survival rates range from over 80% for early-stage cancers to 15% or less for advanced or metastatic tumors [4–6]. Despite advances in computed tomography (CT) screening [7], precision medicine [8], and immunotherapy [9], 65% of NSCLCs and over 90% of SCLCs are currently incurable at presentation.

Despite public health measures to limit the consumption of tobacco products [10], the majority of lung tumors are directly linked to cigarette smoking [11, 12]. In addition, tobacco-associated polycyclic aromatic hydrocarbons (PAH) in urban air pollution have been connected to a dose-dependent rise in the risk of lung cancer and may promote lung cancers (particularly adenocarcinomas) in nonsmokers [13]. In spite of exhaustive research efforts, the genetic and epigenetic mechanisms governing the initiation and spread of tobacco-induced lung cancers remain incompletely understood [14–18].

Rapid advances in transcriptome analysis have identified that over 90% of the human genome is transcribed as noncoding RNAs (Rinn and Chang, 2012). Whereas significant attention has been devoted to understanding transcription and activities of microRNAs (miRs) in normal and cancer cells [19–21], recent evidence indicates that long noncoding RNAs (lncRNAs) are essential in regulating chromatin organization and gene transcription during normal physiological homeostasis and carcinogenesis [22, 23]. LncRNAs, as transcripts more than 200 nucleotides in length, interact with DNA, RNA, and proteins to fine-tune transcriptional and posttranscriptional gene regulation, splicing, and protein stability [24, 25]. Although several lncRNAs, such as HOX transcript antisense RNA (HOTAIR), metastasis-associated lung adenocarcinoma transcript 1 (MALAT1; also known as nuclear-enriched abundant transcript 2—NEAT2), and smoking and cancer-associated lncRNA 1 (SCAL1; also referred to as lung cancer-associated transcript 1—LUCAT1), have been linked to the pathogenesis of lung cancer, the specific mechanisms and clinical advancements of lncRNA dysregulation in these neoplasms have not as yet been fully characterized [26–29]. The current investigation sought to dissect cellular and molecular mechanisms by which lncRNAs contribute to the development of tobacco-related lung cancer.

## Methods

*Cell lines:* All lung cancer cell lines from The American Type Culture Collection (ATCC; Manassas, VA) were kept in RPMI media supplemented with 10% FBS, 10 mM of glutamic acid, and 1% penicillin/streptomycin (normal media). Primary normal human small airway epithelial cells (SAEC) from Lonza, Inc. (Frederick, MD) were cultured following the vendor’s instructions. The immortalized human bronchial epithelial cells (HBEC) generously offered by John D. Minna (U-T Southwestern, Dallas, TX) were cultivated according to the instructions [30]. All cells were routinely checked for mycoplasma using a Sigma kit (cat. no. MP0025) and confirmed by HLA typing to match their original stocks.

*Human tissues:* Under protocols approved by the NIH internal review board (no. 06C0014; dated 02/28/2023), patients undergoing potentially curative resections had their original lung tumor tissues and adjacent lung parenchyma with normal histology collected intraoperatively. Written informed consent was required. All tissues were momentarily frozen right away, and a part of the collected tissue was sent, blindly, to an independent anatomic pathologist for immediate histologic confirmation. Bar-coded tissue specimens were kept in the NCI Thoracic Surgery Branch.

*Murine models:* Four to five-week-old female athymic nude mice were purchased from Charles River Laboratories (Wilmington, MA). The National Cancer Institute Animal Care and Use Committee approved all animal procedures (no. SB-200; dated 07/03/2024).

*Cigarette smoke and drug exposures:* After being prepared as previously described [30], cigarette smoke condensates (CSC) generated from Kentucky Reference 1R4F research blend cigarettes (University of Kentucky) were resuspended in DMSO at a stock concentration of 25 mg tar/ml. Cells were grown in 10-cm plates in suitable normal media (NM) containing DMSO or NM containing CSC (0.025 mg/ml) for smoke exposure experiments. Daily medium changes included the inclusion of brand-new CSC or DMSO control. Cells were collected for analysis at different times after being subcultured as needed. The DNA demethylating agent, decitabine (MilliporeSigma, St. Louis, MO) was added to the culture medium daily (100 nM × 72 h). Mithramycin was obtained from Sigma. Methysticin (MCE) was obtained from MilliporeSigma, St. Louis, MO. Cells were cultivated in NM containing or lacking mithramycin or methysticin for drug exposure treatments. After changing the media and adding mithramycin or methysticin for 24 h at the recommended concentrations, the cells were harvested for additional analysis at the designated times. NFkB-P65 siRNA (#6261S /Cell Signaling) was used to knockdown *NFkB* in relative experiments.

*Arraystar Human lncRNA/mRNA expression analysis:* Total RNA of four samples (SAEC cells with or without CSC exposure; duplicates) was measured with the Nanodrop 1000 and the RNA integrity was evaluated by Agilent 2100 Bioanalyzer. Total RNA (5 μg) from every sample was applied for labeling and array hybridization following these procedures: (1) superscript ds-cDNA synthesis kit from Invitrogen for reverse transcription; (2) one-color DNA labeling kit from NimbleGen for ds-cDNA labeling; (3) NimbleGen hybridization system for array hybridization followed by wash buffer kit from NimbleGen; (4) Axon GenePix 4000B microarray scanner from Molecular Devices Corporation for array scanning. The NimbleScan software (version 2.5) was then used to import the TIFF-formatted scanned images and analyze the expression data for grid alignment. Through the Robust Multichip Average (RMA) algorithm included in the NimbleScan program and quantile normalization, the mRNA level (*_RMA.calls) and Probe level (*_norm_RMA.pair) files were produced. For additional analysis, version 11.0 of Agilent GeneSpring software was used to import the four mRNA-level files. For data analysis, lncRNAs and mRNAs with values that surpass or match the 50.0 lower cut-off (“All Targets Value”) were selected for at least two out of four samples. Fold-change filtering was used to identify mRNAs and lncRNAs that were differently expressed. Pathway analysis and Gene Ontology (GO) analysis were used to determine how these differentially expressed mRNAs function in cancer cells. Finally, hierarchical clustering was carried out to display distinct lncRNA and mRNA expression profiling among samples. Array data have been deposited at the Gene Expression Omnibus (GEO GSE282877).

*Plasmid constructs for overexpression:* Overexpressing constructs of cDNA for *ZFAS1* (pCMV6-AC-ZFAS1;CW303744), cDNA overexpressing constructs for *ZNFX1*(pCMV6-ZNFX1, cat no. RG214589), and vector control for pCMV6 (PS100001 or pCMV6-C-tGF: PS100010 or PCMV6XL4: 496,978) were purchased from OriGene Technologies, Inc.. Using Lipofectamine 2000 (Invitrogen, Carlsbad, CA) was used for  transfection of plasmid constructs or antisense oligos (50 nM or 30 nM respectively);48–72h later, results were analyzed.

*siRNA and shRNA knockdown:* Cells were temporarily or permanently transfected with siRNAs/shRNAs targeting *ZNFX1*(sc-77009, Santa Cruz Biotechnology Inc.), *ZFAS1* (Thermo Fisher), (17–601, Millipore), *NF*κ*B-P65* (6261S, Cell Signaling), or control siRNA/shRNAs (sc-37007, Santa Cruz Biotechnology Inc.; 4,390,844, ThermoFisher; SIC001, Millipore; 6568, Cell Signaling), utilizing Invitrogen Lipofectamine 2000. Target gene knockdown was verified using western blot and qRT-PCR methods.

*Quantitative RT-PCR (qRT-PCR):* Total RNA was extracted for mRNA using TRIzol reagent (Invitrogen) with the elimination of genomic DNA by TURBO DNA-free Kit (Ambion). iScript reverse transcriptase (Bio-Rad) or Invitrogen’s SuperScript™ III first-strand synthesis system was used to reverse transcribe one microgram of total RNA. No addition of reverse transcriptase acted as a negative control. Invitrogen’s Platinum PCR SuperMix was used for the amplification of cDNA. PCR was carried out as follows: 5 min at 94 °C, 35 cycles of 60 s at 94 °C, 60 s at 57–60 °C, and 60 s at 72 °C, followed 5 min at 72 °C. Real-time qRT-PCR analysis was performed as described [31] using ZNFX1, ZFAS1, SP1, and β-actin primers ordered from Applied Biosystems or Integrated DNA Technologies.

*Western blot analysis:* Protein extracts were produced per earlier instructions [31]. Samples were blotted onto Immobilon P membrane (Millipore) and separated on NuPAGE 4–12% Bis–Tris gels (Invitrogen). Proteins were then detected with enhanced chemiluminescence detection reagents (Amersham). Antibodies specific for rabbit anti-SP1 (Santa Cruz Biotechnology), mouse polyclonal anti-ZNFX1 (Abcam), and β-actin antibody (Santa Cruz Biotechnology) were selected and applied for western analysis.

*Proliferation assays/bromodeoxyuridine enzyme-linked immunosorbent assay (BrdU-ELISA):* BrdU ELISA was performed to measure cell proliferation using the CytoSelect™ BrdU Cell Proliferation ELISA kit (Cat#CBA-251, Cell Biolabs, USA). Briefly, cells (2 × 10^4^/well/100 μL) were cultivated in NM containing or lacking specific plasmid constructs or siRNAs in 96-well plates for 0, 24, 48, 72, and 96 h followed by being incubated with 10 µL of 10 × BrdU solution in each well for 4 h at the end of each experimental time point. After washing with PBS, the cells were incubated in 100 µL Fix/Denature Solution at 37 °C for 30 min to fix and denature cellular DNA. Next, the cells were incubated in 100 µL diluted anti-BrdU antibody, followed by horseradish peroxidase (HRP)-coupled secondary antibody diluent. Finally, cell proliferation was evaluated by measuring the absorbance at 450 nm using a microplate reader. Each experiment was performed in triplicate.

*In vitro invasion:* Semipermeable modified Boyden chambers (Millipore, Billerica, MA) coated with extracellular matrix protein (ECM) were used to assess cell invasion in vitro. In the chamber or insert, cells were cultivated at a density of 2.5 × 10^4^ cells/well. Except for serum, the holding well and insert were exposed to the same composition of the medium. While 10% FBS acted as a chemoattractant in the lower well, the insert was devoid of serum. Reagents were applied to the chambers based on the experiment. The cells inside the insert were gently collected using a cotton swab following a 48-h treatment period at 37 °C in a 5% CO_2_ incubator. The manufacturer’s instructions (#ECM551, Millipore, Burlington, MA) were followed for staining and quantifying the cells on the insert’s reverse side.

*Murine xenograft experiments:* A549 and H358 cells with transfection of pCMV6 vector control (or pCMV6-C-tGF: PS100010 or PCMV6XL4: 496,978, Origene, Inc.), or pCMV6-AC-ZFAS1 (CW303744, Origene, Inc.), or pCMV6-ZNFX1(RG214589, Origene, Inc.) were collected in PBS at a concentration of 5 × 10^5^ cells/300 μL for *ZFAS1* OEX and 1 × 10^6^ cells/500 μL for *ZNFX1* OEX, and implanted subcutaneously into opposing flanks of athymic nude mice. Tumor sizes were computed using perpendicular diameters, and the mice were observed twice a week. After about 25 and 30 days, respectively, for the H358 and Calu-6 trials, mice were sacrificed, and the masses of removed xenografts and tumor take percentages were assessed. Xenograft tissues were then cryopreserved in liquid nitrogen and kept for later examination.

*Chromatin immunoprecipitation (ChIP):* Cells cross-linked with 1% formaldehyde were lysed and sonicated on ice to produce DNA fragments that ranged in length from 200 to 800 bp on average. One percent of each sample was retained as the input control following preclearing. EZH2, SUZ12, BMI1, DNMT1, DNMT3A, DNMT3B, H3K4me1, H3K4me3, H3K27ac, H3K27me3, H3K36me3, H3K9me3 (Abcam), RNA polymerase II (Upstate), SP1 (Millipore, Billerica, MA), or IgG control were the specific recognitions used in the immunoprecipitation process. After separating and purifying the DNA from complexes, the target sequences specific in promoters or genomic loci were amplified by PCR using primers listed in Supplementary Table S1 under the prescribed conditions [31].

*Methylated DNA immunoprecipitation assay (MeDIP):* Sonication of extracted genomic DNA resulted in fragments 200–800 bp in size. A typical immunoprecipitation assay for methylated DNA [32] was performed using 5 μg of fragmented DNA, which was incubated with protein A agarose beads after being precipitated with 10 μl monoclonal antibody against 5-methylcytidine (Eurogentec, Seraing, Belgium, http://www.eurogentec.be). DNA was extracted using the phenol–chloroform method and then precipitated with ethanol. Primers for PCR listed in Supplementary Table S1 were designed specifically within 2000 bp upstream of the *ZNFX1* transcription start site and enhancer region of *ZFAS1*; amplicon ranged in size from 200 to 350 bp.

*Bisulfite sequencing:* Using a QIAGEN Epifect kit (QIAGEN), genomic DNA was treated with bisulfite. According to instructions, the PCR products were isolated from agarose gels, then purified and subcloned as indicated [31]. Supplementary Table S1 lists primers specific for bisulfite sequencing.

*Methylation-specific PCR (MSP):* Using a QIAGEN Epifect kit (QIAGEN), genomic DNA was bisulfite treated. To perform the MSP analysis, promoters of the genes were identified using an online data analysis tool called MethPrime (https://genome.ucsc.edu). The methylated and unmethylated primers (Supplementary Table S1) were designed using MethPrime online software and the preferred sequences. The PCR products were analyzed and visualized using ethidium bromide staining and electrophoresis in 2% agarose gel.

*RNA cross-link immunoprecipitation:* Cells were harvested, and CLIP assays were performed using either anti-PRC family or DNMTs antibodies as described [31]. In short, cells were lysed to produce RNA/protein (PRC/DNMT) complexes after being cross-linked using 400 mJ/cm^2^ of radiation and an extra 200 mJ/cm^2^ in Stratalinker [33, 34]. Of each sample, 1% was retained as the input fraction following precleaning. Immunoprecipitation was executed with specific antibodies against polycomb proteins, DNMTs, or control IgG (Supplementary Table S2). PCR amplification of target regions was followed by extraction and purification of RNA from the complexes.

*Formaldehyde-assisted isolation of regulatory elements (FAIRE):* FAIRE was used to characterize the upstream regulatory element for *ZFAS1*. Briefly, cultured cells were directly treated with formaldehyde. The chromatin after cross-linkage was sheared by sonication and separated with phenol–chloroform extraction. The chromatin crosslinking profile is most likely to be dominated by cross-linkage between histones and DNA or between one histone and another [35, 36]. Only DNA fragments devoid of proteins remain in the aqueous phase after covalently bound DNA–protein complexes are sequestered in the organic phase. The same procedure for the hybridization reference is performed on the cells that had not been fixed with formaldehyde, a procedure identical to a traditional phenol–chloroform extraction. All the isolated and purified genomic DNA was analyzed by PCR with genomic site-specific primers as shown in Supplementary Table S1. Quantitative FAIRE assay revealed the only genomic region specific for the enrichment of H3K4me1/H3K27ac and CpG island position is the putative enhancer for *ZFAS1* (Fig. 8A).

*Bioinformatic analysis:* Gene expression correlation analysis of publicly available scRNA-seq datasets (reference to PMID: 37,910,161) was performed using R (version 4.1.2) to examine the relationship between *ZFAS1* and *ZNFX1* expression across different epithelial cell types and smoking status groups. Pearson correlation coefficients and corresponding *p*-values were calculated using the cor.test function. The analysis was conducted both globally across all cells and stratified by cell type and smoking status (never smoker versus smoker). Correlation plots were generated using R’s base plotting system, with separate visualizations for the entire cell population and smoking status subgroups. Linear regression lines were added to each scatter plot to illustrate the relationship trends. For cell type distribution analysis, to analyze the distribution of epithelial cell types between smoking status groups, we created contingency tables using R’s table function. Cell types were ordered by their total frequency in descending order to facilitate interpretation. The distribution was visualized using a stacked barplot, where each bar represents a distinct cell type, and the segments within each bar represent the proportion of cells from never smokers (light blue) and smokers (salmon). Cell counts were directly annotated on the bars for each smoking status group. Both analyses utilized the same single-cell RNA sequencing dataset, with cell type classifications stored in the “EPIClass” metadata column and smoking status information in the “Smoking” column of the Seurat object. All statistical analyses and visualizations were performed in R, leveraging the Seurat framework for single-cell data handling. Data resources were published as “Nakayama , and Yamamoto Y. Cancer-prone phenotypes and gene expression heterogeneity at single-cell resolution in cigarette-smoking lungs” [37].

*Quantification and statistical analysis:* The data are shown as mean ± SEM. The signed-rank test was used to assess variations between lung cancer patients’ matched tumors and normal tissues. We used the Wilcoxon–Mann–Whitney test to compare the groups. The bias-corrected and accelerated bootstrapping methods were utilized to calculate the confidence intervals for the different medians between the two groups. Five-day intervals were used to assess variations in tumor volumes among groups of tumor-bearing mice using the Wilcoxon signed-rank test, which was also applied to calculate exact nonparametric confidence intervals with two tails for the flank difference median. P values underwent total resampling to account for multiple tests. *p* < 0.05 was regarded as significant.

*Data and software availability:* The dataset from the Arraystar array will be deposited, and the accession number will be available soon.

*Key Resources tables:* Supplementary Tables S1 and S2, respectively, contain a list of the primers and antibodies used in this investigation.

## Results

*Effects of cigarette smoke on ZFAS1 and ZNFX1 expression in normal respiratory epithelia and lung cancer cells.* Preliminary Arraystar array experiments were conducted to examine lncRNA and mRNA expression in SAEC cells cultured in NM with DMSO (control) or CSC plus DMSO for 5 days. CSC exposure modulated the transcription activities of numerous lncRNAs and protein-coding genes (Supplementary Fig. S1A, B). Subsequently, association analysis of coincident alterations was performed, focusing on lncRNAs and genes encoding proteins located on the same chromosome within a distance of less than 0.5 million base pairs, resulting in the identification of 13 pairs of lncRNAs and genes encoding proteins (Supplementary Fig. S1C). Notably, *lncRNA-zinc finger NFX1-type containing 1 antisense RNA 1 (ZFAS1)* and *zinc finger NFX1-type containing 1 (ZNFX1)* were divergently transcribed with their transcription start sites (TSSs) immediately adjacent to each other (Supplementary Fig. S1D), implying the potential existence of a *cis*-acting regulatory correlation between *ZFAS1* and *ZNFX1*. Microarray data revealed that CSC increased *ZFAS1* expression by approximately 2.5-fold while decreasing *ZNFX1* expression by approximately 3.6-fold in SAEC (Supplementary Fig. S1C, E).

**Fig. 1:**
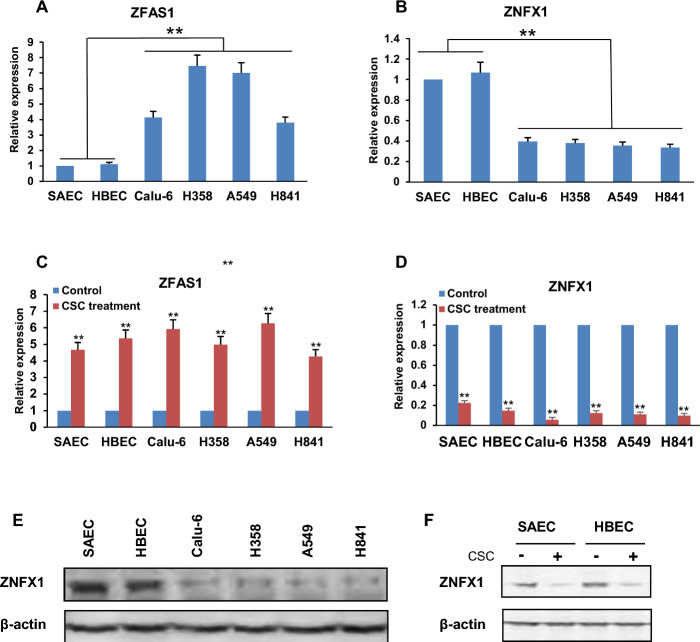
CSC modulates reciprocal transcriptional activities of *ZFAS1* and *ZNFX1* in normal respiratory epithelia and lung cancer cells. **A**, **B** qRT-PCR analysis demonstrating endogenous ZFAS1 (**A**) and ZNFX1 (**B**) mRNA levels are significantly higher or lower, respectively, in SAEC and HBEC cells relative to Calu-6, H358, A549, and H841 cells. **C**, **D** qRT-PCR analysis demonstrating upregulation of *ZFAS1* (**C**) with concomitant downregulation of *ZNFX1* (**D**) in SAEC, HBEC, Calu-6, H358, A549, and H841 cells following 5-day CSC exposure. * *p* < 0.05; **p < 0.01. **E** Immunoblot analysis demonstrates that endogenous ZNFX1 protein levels are higher in SAEC and HBEC cells compared with Calu-6, H358, A549, and H841 cells. **F** Immunoblot demonstrating decreased ZNFX1 protein levels in SAEC and HBEC following 5-day CSC exposure. Data are mean ± SEM; *T*-test; *n* = 3; * *p* < 0.05; **p < 0.01

To confirm and extend these observations, qRT-PCR and immunoblot assays were conducted to examine *ZFAS1* and *ZNFX1* expression in SAEC as well as cdk-4/h-TERT immortalized HBEC, and Calu-6, H358, A549, and H841 NSCLC lines cultivated in the presence or absence of CSC for 5 days. Those cell lines were selected on the basis of their relevance to the research question and their well-documented association with smoking-related cancer types. They are widely used in the scientific community for studies on smoking-related carcinogenesis and cancer biology, as they represent various stages of tumor progression and mimic the molecular characteristics observed in smokers and nonsmokers alike. Untreated Calu-6, H358, A549, and H841 cells exhibited higher endogenous ZFAS1 and lower endogenous ZNFX1 mRNA levels relative to SAEC and HBEC (Fig. 1A, B). CSC exposure upregulated *ZFAS1 *expression approximately 4.7–5.4-fold in SAEC and HBEC; similar treatment increased *ZFAS1* expression approximately 4.3–6.3-fold in Calu-6, H358, A549, and H841 cells (Fig. 1C). Additionally, CSC exposure downregulated *ZNFX1* expression approximately 4.5–6.7-fold in SAEC and HBEC, and decreased *ZFAS1* expression approximately 8.2–17.7-fold in Calu-6, H358, A549, and H841 cells, respectively (Fig. 1D). Immunoblot analysis (Fig. 1E; Supplementary Fig. S2) demonstrated that ZNFX1 protein levels were much lower in lung tumor cells relative to SAEC and HBEC. CSC exposure decreased ZNFX1 protein levels in SAEC and HBEC (Fig. 1F; Supplementary Fig. S2). These effects could not be convincingly demonstrated in lung cancer lines owing to extremely low endogenous ZNFX1 protein levels in these cells (data not shown).

**Fig. 2:**
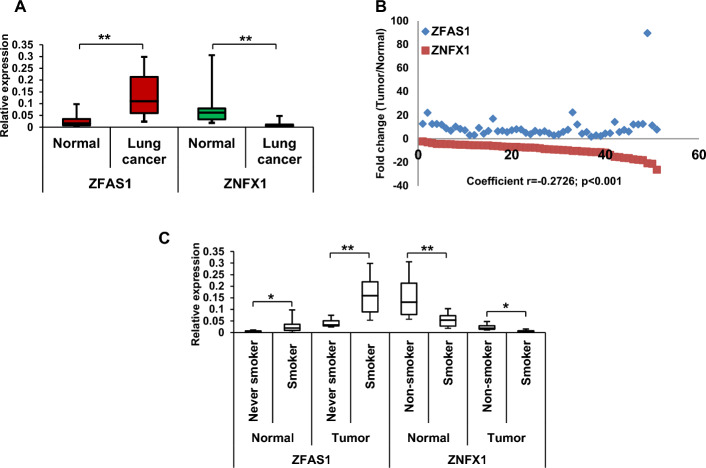
Reverse correlation between expression of *ZNFX1* and  *ZFAS1* in lung cancer specimens. **A** qRT-PCR analysis of ZFAS1 and ZNFX1 mRNA levels in 51 lung cancer specimens and their paired adjacent normal lung tissues. **B** Correlation patterns and corresponding correlation coefficient values between ZFAS1 and ZNFX1 in 51 lung cancers. Coefficient *r* = −0.2726, *p* < 0.001. **C** qRT-PCR analysis of *ZFAS1* and *ZNFX1* expression in lung cancers from smokers/former smokers versus never smokers. * *p* < 0.05; **p < 0.01

*ZFAS1 and ZNFX1 expression in primary specimens of lung cancer.* To determine if alterations in *ZFAS1* and* ZNFX1* expression were potentially relevant to pulmonary carcinogenesis, we performed qRT-PCR experiments to evaluate *ZFAS1* and *ZNFX1* activation in a randomly selected panel of 51 primary NSCLC and adjacent lung tissues with normal histology from 42 smokers/former smokers and 9 never smokers. As shown in Fig. 2A, ZFAS1 mRNA levels were higher  (mean ~ 5.9-fold; range 3.0–22.2-fold) in lung tumors compared with paired adjacent normal lung tissues (p < 0.01). In contrast, ZNFX1 mRNA levels were lower (mean 7.4-fold; range 6.4–12.1-fold) in lung cancers relative to paired normal lung tissues (p < 0.01). *ZFAS1* expression was negatively correlated with *ZNFX1* expression in tumors (*r* = −0.2726) from all 51 paired samples (Fig. 2B). The magnitude of *ZFAS1* overexpression and ZNFX1 downregulation was more significant in lung tumors from active and former smokers compared with never smokers (3.7 fold higher than never smokers for *ZFAS1* versus 3.9 fold lower than never smokers for *ZNFX1*, respectively; *p* < 0.01; Fig. 2C). In addition, ZFAS1 mRNA levels were elevated whereas ZNFX1 mRNA levels were reduced in lung parenchyma with normal histology from smokers/former smokers in comparison with never smokers (Fig. 2C), suggestive of a field effect.

Given the limited smoking-related lung cancer specimens for analysis of the inverse correlation of *ZFAS1* and *ZNFX1 *and the complexity of cell populations of these specimens, we performed additional correlation analysis using publicly accessible scRNA datasets [37]. We found that smoking history induced an enhanced negative correlation of ZFAS1 and ZNFX1 in all epithelia (nonsmokers: df = 53,754, *p*-value < 5.14 × 10^−52^, coefficient = − 0.06568549 versus smokers: df = 24,082, *p*-value < 6.44 × 10^−37^, coefficient = −0.08165988) (Supplementary Fig. S3A–C). For cell-type specific analysis, the negative correlations of ZFAS1 and ZNFX1 were variably impacted by smoking exposure in these 13 different cell types (total population), in which more negative coefficients were identified from smokers than never smokers in AT1, AT2, and Club cells, which were highly significant(Supplementary Fig. S3B, C). This analysis of of normal versus smoke-exposed lungs at a single-cell level provides further evidence and deeper insights into the potential clinical relevance of the relationships between *ZFAS1* and *ZNFX1* expression during pulmonary carcinogenesis.

**Fig. 3:**
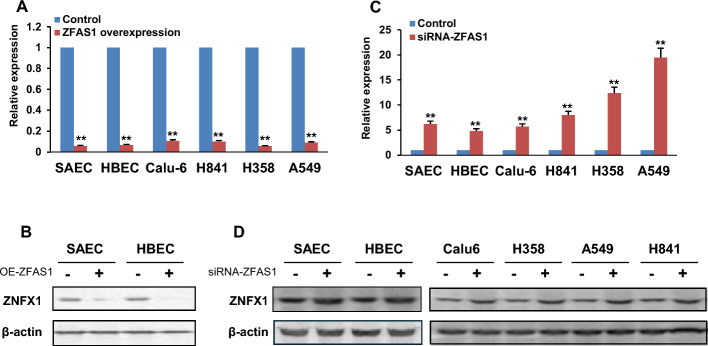
ZFAS1 directly antagonizes transcriptional activity of *ZNFX1* in normal respiratory epithelia and lung cancer cells. **A** qRT-PCR analysis demonstrating that overexpression of *ZFAS1* downregulates *ZNFX1* in normal respiratory epithelia and lung cancer cells. **B** Immunoblotting analysis demonstrating that overexpression of *ZFAS1* reduces ZNFX1 protein levels in SAEC and HBEC. **C** qRT-PCR analysis demonstrating that knockdown of *ZFAS1* upregulates *ZNFX1* in normal respiratory epithelia and lung cancer cells. **D** Immunoblot demonstrating that depletion of ZFAS1 increases ZNFX1 protein levels in normal respiratory epithelia and lung cancer cells. Data are mean ± SEM; *t*-Test; *n* = 3; * *p* < 0.05; **p < 0.01

*Effects of ZFAS1 on transcriptional activity and function of ZNFX1 in normal respiratory epithelia and lung tumor cells.* We next performed experiments to ascertain if *ZFAS1* directly regulates *ZNFX1* transcription in respiratory epithelial cells. Briefly, *ZFAS1* was constitutively overexpressed or knocked-down using siRNA techniques or antisense oligos in SAEC, HBEC, Calu-6, H841, H358, and A549 cells. Preliminary qRT-PCR experiments confirmed overexpression or knockdown of *ZFAS1* in all cell lines relative to respective vector controls (Supplementary Fig. S4A, B).). Subsequent qRT-PCR experiments demonstrated marked downregulation of *ZNFX1* in normal respiratory epithelia and lung malignant cells overexpressing *ZFAS1* (Fig. 3A). Immunoblot experiments (Fig. 3B; Supplementary Fig. S4C) confirmed that *ZFAS1* overexpression decreased ZNFX1 protein levels in SAEC and HBEC; this phenomenon could not be demonstrated in lung cancer cells owing to very low levels of ZNFX1 in control cells (data not shown). In contrast, knockdown of *ZFAS1* increased ZNFX1 mRNA and protein levels in normal pulmonary epithelia and lung tumor cells (Fig. 3C, D; Supplementary Fig. S4D).

**Fig. 4:**
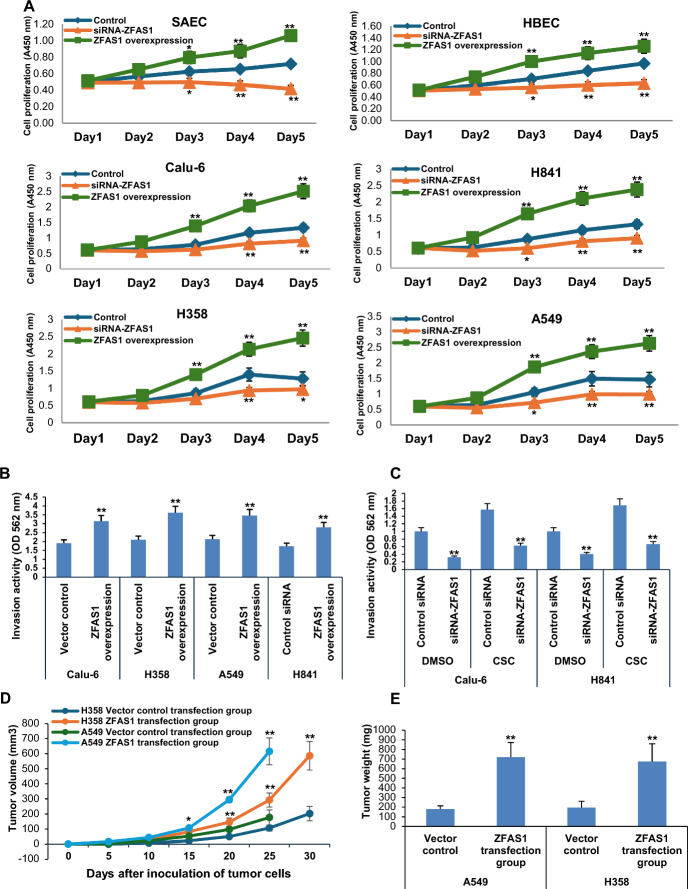
*ZFAS1* functions as an oncogene in lung cancer cells. **A** Effects of *ZFAS1* expression on in vitro proliferation of SAEC and HBEC, as well as Calu-6, H841, H358, and A549 lung cancer cells. *ZFAS1* promotes cell growth in normal respiratory epithelia and lung tumor cells. **B**, **C** Matrigel invasion assays demonstrate that overexpression of *ZFAS1* enhances invasion of Calu-6, H358, A549, and H841 lung cancer cells, whereas siRNA knockdown of *ZFAS1* inhibits invasion of control lung cancer cells and attenuates CSC-mediated invasion potential in these cells. *p < 0.05; **p < 0.01. **D** Growth of H358 and A549 subcutaneous xenografts in nude mice. Volumes of xenografts derived from H358 and A549 cells overexpressing *ZFAS1* are significantly larger than control xenografts. **E** Tumor masses from H358 and A549 xenografts. *ZFAS1* overexpression significantly increases the average mass of tumor xenografts. Data are mean ± SEM; *T*-test; *n* = 3; * *p* < 0.05; **p < 0.01

*ZFAS1 is an oncogene in human lung tumor cells.* We next performed experiments to examine if ZFAS1 modulates the phenotype of lung malignant cells. First, we performed in vitro proliferation assays using SAEC, HBEC, Calu-6, H841 H358, and A549 cells following constitutive overexpression or knockdown of *ZFAS1*. Overexpression of *ZFAS1* increased growth of SAEC, HBEC, Calu-6, H841, H358, and A549 cells compared with respective vector controls; in contrast, knockdown of *ZFAS1* inhibited proliferation of these cells (Fig. 4A). The effects of *ZFAS1* overexpression were particularly dramatic, whereas the inhibitory effects of depletion of this lncRNA were relatively modest in lung tumor cells, possibly owing to different levels of *ZFAS1* expression, relative efficiencies of gene manipulation, and/or activation of pathways that can compensate for loss of *ZFAS1* expression in these cells.

We next conducted Matrigel assays to assess the impact of lnc-RNA ZFAS1 on invasion potential of lung tumor cells. Overexpression of *ZFAS1* increased invasion capacities of Calu-6, H358, A549, and H841 cells (Fig. 4B). Consistent with previously published findings [31], CSC enhanced invasion potential in lung tumor cells (Fig. 4C). Knockdown of *ZFAS1* significantly decreased invasion of lung cancer cells and attenuated the enhancement effect of CSC in these cells (results for Calu-6 and H841 cells are described in Fig. 4C).

We next examined if *ZFAS1* modulates proliferation of lung tumor cells in-vivo. H358 and A549 cells with stable overexpression of *ZFAS1* or control vectors were implanted subcutaneously in both opposing flanks of athymic nude mice (8–10 tumors per group). Masses and volumes of tumor xenografts established from lung cancer cells constitutively expressing *ZFAS1* were significantly larger than those from control cells (Fig. 4D, E; *p* < 0.01).

**Fig. 5:**
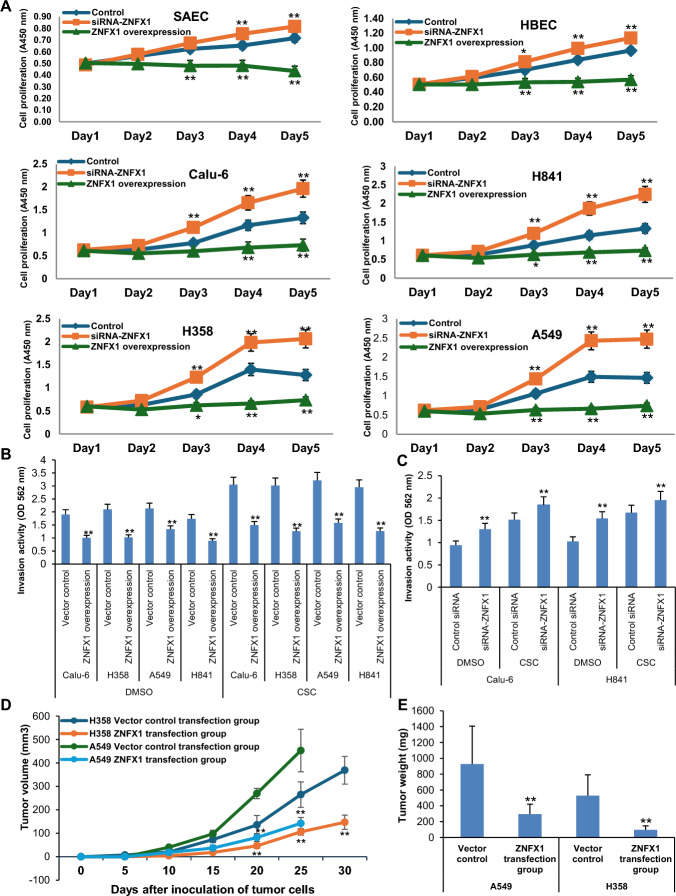
*ZNFX1* functions as a tumor suppressor in lung cancer cells. **A** Effects of *ZNFX1* expression on in vitro proliferation in SAEC and HBEC as well as Calu-6, H841, H358, and A549 cells. *ZNFX1* promotes cell growth in both normal respiratory epithelia and lung tumor cells. **B**, **C** Matrigel invasion assays demonstrating that overexpression of *ZNFX1* inhibits, whereas knockdown of *ZNFX1* increases invasion of Calu-6 and H841 lung cancer cells. Knockdown of *ZNFX1* enhances CSC-induced invasion of lung cancer cells. * *p* < 0.05; ** *p* < 0.01. **D** Growth of H358 and A549 subcutaneous xenografts in nude mice. Volumes of xenografts derived from H358 and A549 cells overexpressing ZNFX1 are significantly smaller than control xenografts. **E** Tumor masses from H358 and A549 xenografts. *ZNFX1* overexpression significantly decreases the average mass of tumor xenografts. Data are mean ± SEM; T-test; *n* = 3; * *p* < 0.05; **p < 0.01

*ZNFX1 is a tumor suppressor in lung cancer cells.* We next examined the effects of *ZNFX1* expression in normal respiratory epithelia as well as lung tumor cells. cDNA plasmid constructs and siRNAs were used to overexpress or knock down* ZNFX1* in SAEC and HBEC, as well as Calu-6, H358, A549, and H841 cells. Preliminary qRT-PCR and immunoblot assays confirmed  overexpression or knockdown of *ZNFX1* in these cells (Fig. S5;A-D). We then performed in vitro proliferation assays using SAEC, HBEC, Calu-6, H841, H358, and A549 cells following constitutive overexpression or knockdown  of *ZNFX1*. Overexpression of *ZNFX1* virtually abolished proliferation of SAEC and HBEC, as well as all four lung cancer lines (Fig. 5A). In contrast, knockdown of *ZNFX1* modestly enhanced proliferation of SAEC and HBEC; even though endogenous levels of *ZNFX1* expression were quite low in lung cancer lines, knockdown of *ZNFX1 *enhanced growth of lung cancer cells (Fig. 5A). Additionally, constitutive overexpression of *ZNFX1* decreased Matrigel invasion in Calu-6, H358, A549, and H841 cells, and markedly arrested CSC mediated augmentation of invasion of these cells (Fig. 5B). In contrast, knockdown of *ZNFX1* increased invasion of Calu-6 and H841 lung tumor cells (Fig. 5C), and modestly but significantly increased invasion of these cells when exposed to CSC (Fig. 5C).

We next investigated the impact of *ZNFX1* expression on the in vivo growth of lung cancer cells. Xenografts established from A549 and H358 lung tumor cells with constitutive *ZNFX1* expression had significantly smaller volumes and masses compared with tumors derived from control cells (Fig. 5D, E; *p* < 0.01).

**Fig. 6:**
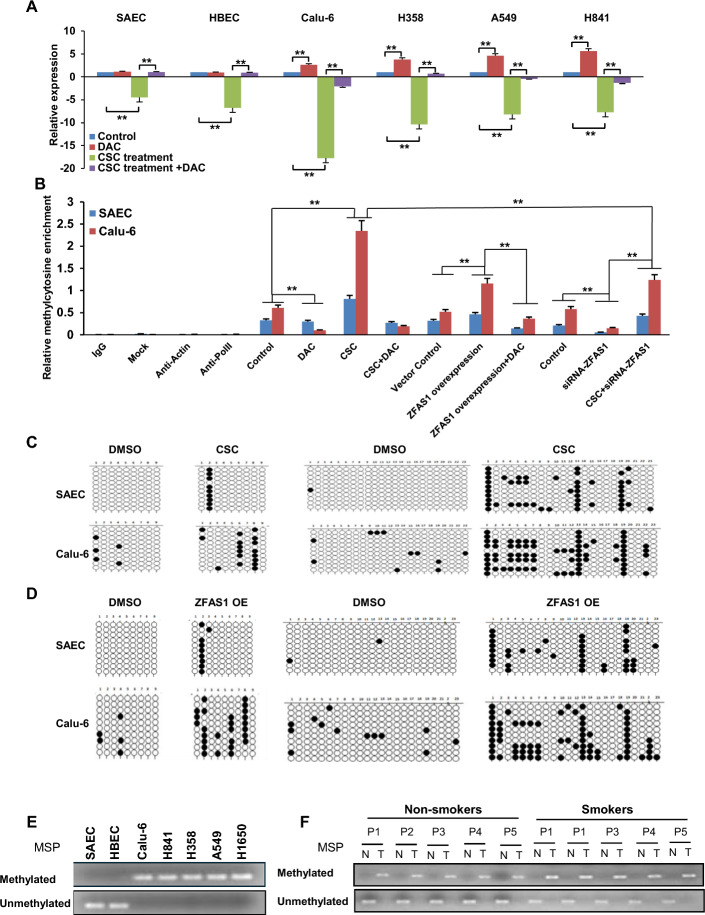
Promoter CpG island-specific methylation alterations coincide with the repression of *ZNFX1*. **A** qRT-PCR analysis demonstrating that DAC increases *ZNFX1* expression in lung cancer cells not in normal lung epithelia. DAC abrogates CSC-mediated repression of *ZNFX1* in normal lung epithelia and lung cancer cells. **B** MeDIP analysis of DNA methylation profiles in the first CpG island proximal to the TSS of *ZNFX1* in SAEC and Calu-6 cells; DAC decreases CSC- or ZFAS1-mediated DNA hypermethylation. **C**, **D** Bisulfite sequencing of two genomic regions in the first CpG island of the *ZNFX1* promoter, demonstrating that CSC or *ZFAS1* overexpression enhances DNA methylation in this CpG island in SAEC and Calu-6 cells. **E** MSP demonstrating site-specific DNA methylation in the *ZNFX1* promoter in lung cancer cells but not in NREC. **F** MSP analysis demonstrating DNA methylation in the first CpG island of the *ZNFX1* promoter in lung cancer tissues from both smokers and nonsmokers relative to normal adjacent lung tissues. Data are mean ± SEM; *T*-test; *n* = 3; * *p* < 0.05; **p < 0.01

*Epigenetic regulation of ZNFX1 expression by CSC-mediated ZFAS1 activation.* We next explored potential mechanisms by which ZFAS1 modulates *ZNFX1* expression. In silico analysis demonstrated three CpG islands within a 1.5 kb region overlapping the *ZNFX1* promoter (Supplementary Fig. S6A, B), suggesting potential epigenetic regulation of *ZNFX1* transcription. qRT-PCR experiments demonstrated that 5-aza-2′ deoxycytidine (DAC);100 nM for 72 h), upregulated *ZNFX1* in lung tumor cells while markedly abrogating CSC or *ZFAS1* overexpression-mediated *ZNFX1* repression in NREC and lung cancer cells (Fig. 6A; Supplementary Fig. S7). Consistent with these findings, MeDIP assays demonstrated that CSC exposure and* ZFAS1* overexpression enhanced DNA methylation within the first CpG island in the *ZNFX1* promoter (region A in Supplementary Fig. S6B; Fig. 6B, Supplementary Fig. S8) in SAEC and Calu-6 cells; notably, DNA methylation induced by CSC or *ZFAS1* overexpression was more pronounced in Calu-6 cells, reflecting increased plasticity relative to normal airway epithelial cells, as we have noted in previous studies [38]. This phenomenon was not observed following analysis of the second CpG island (region B in Supplementary Fig. S6B, C); the third island (region C in Supplementary Fig. S6B) was not evaluated owing to its distance from the *ZNFX1* TSS. DAC abrogated CpG methylation induced by CSC as well as overexpression of *ZFAS1* in SAEC and Calu-6 cells (Fig. 6B). Knockdown of *ZFAS1* also decreased DNA methylation and attenuated CSC-mediated induction of DNA methylation in this region (Fig. 6B). Sodium bisulfite sequencing analysis of two regions within the first CpG island confirmed that CSC exposure increased DNA methylation within the *ZNFX1* promoter in SAEC and Calu-6 cells (Fig. 6C); *ZFAS1* overexpression induced remarkably similar DNA methylation effects (Fig. 6D). Subsequent MSP analysis using primers interrogating the region evaluated by MeDIP experiments demonstrated DNA hypermethylation within the first CpG island in lung cancer lines but not SAEC or HBEC (Fig. 6E). Additional MSP experiments revealed elevated DNA methylation in all 51 lung cancer specimens compared with paired adjacent normal lung tissues (Fig. 6F; Supplementary Fig. S9). While precise quantification was not possible, MSP products seemed to be more noticeable in tobacco-related lung cancers compared with those from never smokers.

**Fig. 7:**
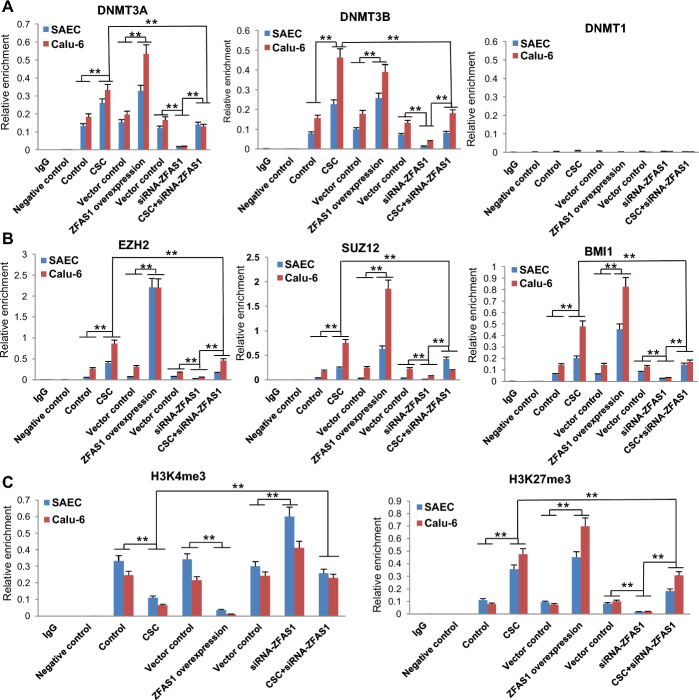
CSC-mediated activation of *ZFAS1* coincides with epigenetic alterations in the promoter of *ZNFX1*. **A** qChIP analysis of DNMT3A, DNMT3B, and DNMT1 levels within the first CpG island of the *ZNFX1 *promoter in SAEC and Calu-6 cells exposed to NM or CSC, following overexpression or knockdown of *ZFAS1*. **B** qChIP analysis of EZH2, SUZ12, and BMI1 levels within the promoter of *ZNFX1* in SAEC and Calu-6 cells either exposed to NM or CSC following overexpression or knockdown of *ZFAS1*. **C** qChIP analysis of H3K4me3 or H3K27me3 within the promoter of *ZNFX1* in SAECs and Calu-6 cells exposed to NM or CSC following overexpression or knockdown of *ZFAS1*. CSC and *ZFAS1* decrease levels of H3K4me3 while increasing H3K27me3 levels in the proximal promoter region (0 to −1 kb) of *ZNFX1* in SAEC and Calu-6 cells; depletion of *ZFAS1* partially abrogates CSC-induced alterations in these histone marks within this region. Data are mean ± SEM; T-test; *n* = 3; * *p* < 0.05; **p < 0.01

We conducted quantitative chromatin immunoprecipitation (qChIP) experiments to further investigate epigenetic mechanisms regulating *ZNFX1* expression in normal respiratoy epithelial cells and lung cancer cells. Upon CSC exposure or *ZFAS1* overexpression, there was a significant increase in occupancy of DNMT3A and DNMT3B, but not DNMT1 within the first CpG island of the* ZNFX1* promoter in both SAEC and Calu-6 cells (Fig. 7A). These changes coincided with recruitment of EZH2, SUZ12, and BMI1, core components of polycomb repressor complexes (PRC) 1 and 2 (Fig. 7B). Recruitment of DNMTs and polycomb proteins coincided with decreased levels of the histone activation mark, H3K4me3 with concomitant increases in the repressive histone mark, H3K27me3 within the proximal promoter region (0 to −1 kb) of *ZNFX1* (Fig. 7C). Knockdown of *ZFAS1* decreased occupancy of DNMT3A, DNMT3B, EZH2, SUZ12, BMI1, and H3K27me3, while increasing H3K4me3 levels in the *ZNFX1* promoter; additionally, knockdown of *ZFAS1* attenuated CSC-mediated alterations in chromatin structure within this region (Fig. 7A–C).

**Fig. 8:**
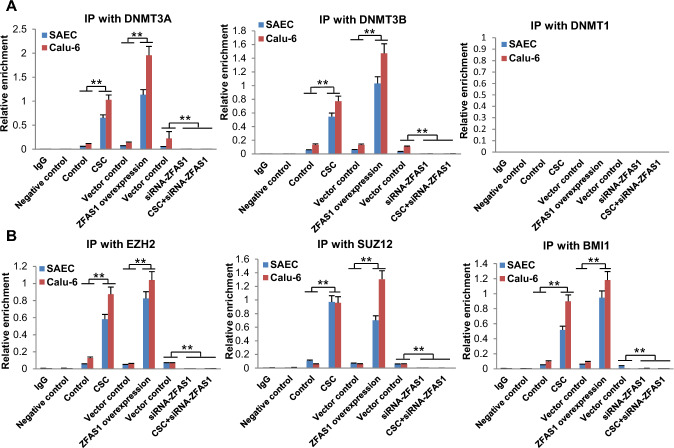
Interactions of ZFAS1 transcripts with DNA methylation and polycomb group proteins. **A**, **B** qCLIP analyses of interactions of ZFAS1 with DNMT3A or DNMT3B and DNMT1 as well as EZH2, SUZ12, BMI1 in SAEC and Calu-6 cells exposed to NM or CSC, with or overexpression or knockdown of *ZFAS1*. CSC and *ZFAS1* over-expression induce binding of DNMT3A, DNMT3B, EZH2, SUZ12, and BMI1 but not DNMT1 to ZFAS1 transcripts; knockdown of *ZFAS1* diminishes these interactions. Data are mean ± SEM; T-test; *n* = 3; * *p* < 0.05; ** *p* < 0.01

Because lncRNAs can recruit epigenetic modifiers to DNA [39, 40], we next performed quantitative RNA cross-link immunoprecipitation (CLIP) experiments to ascertain if ZFAS1 interacts with DNMTs and polycomb proteins. Briefly, DNMT and polycomb proteins were immunoprecipitated from SAEC and Calu-6 cells followed by qRT-PCR amplification of ZFAS1. Preliminary qRT-PCR analysis confirmed the efficiency of overexpression or knockdown of *ZFAS1* in NREC and lung tumor cells (Supplementary Fig. S4). CSC exposure as well as *ZFAS1* overexpression increased the interaction of ZFAS1 with DNMT3A, DNMT3B, EZH2, SUZ12, and BMI1 (Fig. 8A, B). Knockdown of *ZFAS1* abolished CSC-mediated interaction of ZFAS1 with these repressive proteins (Fig. 8A, B).

**Fig. 9:**
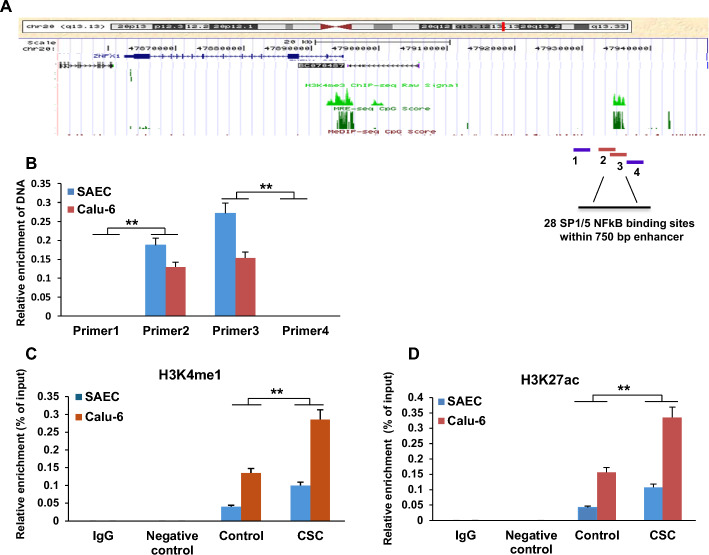
Enhancer-specific modulation of *ZFAS1* by CSC. **A** Schematic depiction demonstrating putative enhancer-like regulatory element region for *ZFAS1*. The numbers 1, 2, 3, and 4 indicate the primer positions for the formaldehyde-assisted isolation of regulatory elements (FAIRE) assay to characterize upstream regulatory elements of *ZFAS1*. **B** Quantitative FAIRE analysis for mapping the regulatory elements of *ZFAS1* identified significant specific amplification signals in primer 2 and 3 positions compared with negative signals from primer 1 and 4 sites, demonstrating that the narrow genetic region (40–41 kb upstream from TSS of *ZFAS1*) is the potential enhancer for *ZFAS1*. **C**, **D** qChIP analysis demonstrating increased levels of H3K4me1 and H3K27ac (histone activation marks) in the putative enhancer of *ZFAS1* in SAEC and Calu-6 cells following CSC exposure. Data are mean ± SEM; T-test; *n* = 3; * *p* < 0.05; ** *p* < 0.01

*CSC modulates transcription of ZFAS1 via its downstream enhancer.* ZFAS1 is a recently annotated lncRNA located on chromosome 20 (USCS genome browser dataset). Genetic and epigenetic environments around *ZFAS1 *from the UCSC database, including the coexistence of a H3K27ac/H3K4me1 peak and a CpG island suggested that a narrow region (40-41 kb upstream from the TSS of *ZFAS1*) contains the specific regulatory DNA elements for this gene (Fig. 9A). Quantitative FAIRE assays confirmed that the only genomic region specific for the H3K27ac / H3K4me1 enrichment peak coexisted with a potential CpG island (Fig. 9B). qChIP experiments demonstrated increased levels of H3K4me1 and H3K27ac in the regulatory region of ZFAS1 in SAEC and Calu-6 cells following CSC exposure (Fig. 9C, D). However, upregulation of *ZFAS1* by CSC did not coincide with DNA demethylation within the CpG island associated with this lncRNA in SAEC or lung cancer cells (S6C, E).

**Fig. 10:**
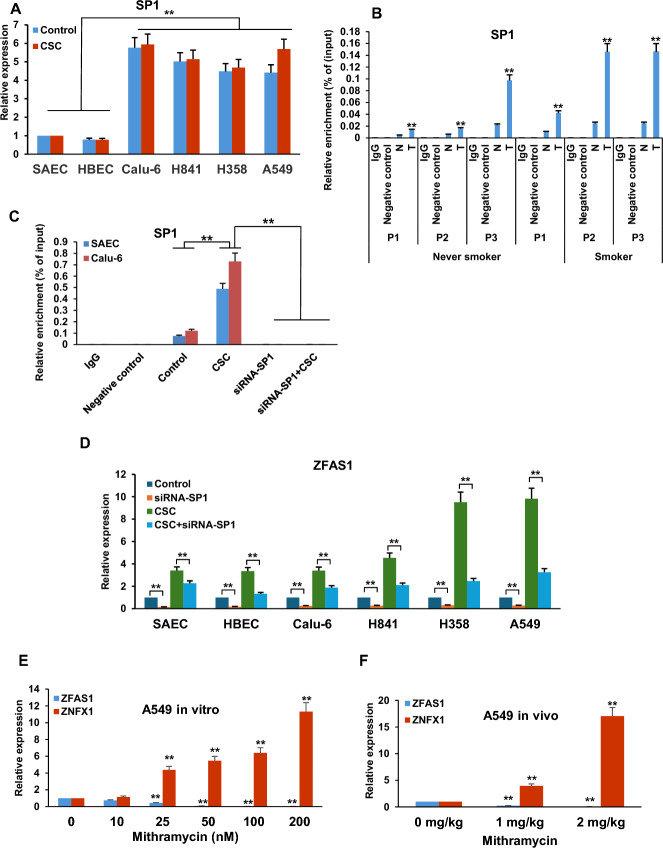
Role of SP1 in CSC-mediated regulation of *ZFAS1*. **A** qRT-PCR analysis of S1  *SP1* expression in NREC and lung cancer cells.* SP1* expression is much higher in lung cancer cells compared with NREC. Short term CSC exposure does not appear to increase *SP1* expression in these cells. **B** qChIP analysis of SP1 occupancy in the *ZFAS1* enhancer in lung cancers and paired normal adjacent lung tissues from smokers and nonsmokers. **C** qChIP analysis demonstrating that CSC exposure induces recruitment of SP1 to the putative enhancer of *ZFAS1* in SAEC and Calu-6 cells. **D** qChIP analysis demonstrating that knockdown of *SP1* decreases endogenous *ZFAS1* expression and markedly attenuates CSC-mediated upregulation of *ZFAS1* in NREC and lung cancer cells. **E**, **F** qRT-PCR analysis demonstrating that mithramycin mediates dose-dependent reduction of *ZFAS1* with a concomitant increase in *ZNFX1* expression in A549 lung cancer cells in vitro (**E**) and in vivo (**F**). Data are mean ± SEM; T-test; *n* = 3; * *p* < 0.05; ** *p* < 0.01

*SP1 contributes to CSC-mediated upregulation of ZFAS1.* Software-guided analysis revealed multiple putative binding sites for Specificity Protein 1 (SP1; 28 sites within the enhancer for *ZFAS1*; Fig. 9A). Given that we have previously demonstrated that SP1 mediates upregulation of the potential stem cell marker, *ABCG2* in lung tumor cells following CSC exposure [41], we questioned if SP1 also contributes to CSC-mediated activation of *ZFAS1*. qRT-PCR assays demonstrated that endogenous levels of SP1 mRNA were higher in lung cancer cells compared with NREC; consistent with our previous observations in esophageal cancer [42], cigarette smoke did not increase *SP1* expression in lung cancer cells (Fig. 10A). qChIP experiments demonstrated increased occupancy of SP1 within the *ZFAS1* enhancer in primary lung tumors relative to paired normal lung tissues (Fig. 10B). Additional qChIP experiments demonstrated that CSC exposure induced recruitment of SP1 to the *ZFAS1* enhancer in SAEC and Calu-6 cells (Fig. 10C). Knockdown of *SP1* decreased basal expression of *ZFAS1*, and significantly attenuated CSC-mediated upregulation of this lncRNA in NREC and lung cancer cells (Fig. 10D; Supplementary Fig. S10). In line with these results, mithramycin (MM), an antineoplastic agent that blocks the binding of SP1 to GC-rich DNA [43], induced dose-dependent downregulation of *ZFAS1* with concomitant upregulation of *ZNFX1 *in cultured A549 lung tumor cells (Fig. 10E), as well as subcutaneous A549 xenografts in athymic nude mice (Fig. 10F).

**Fig. 11:**
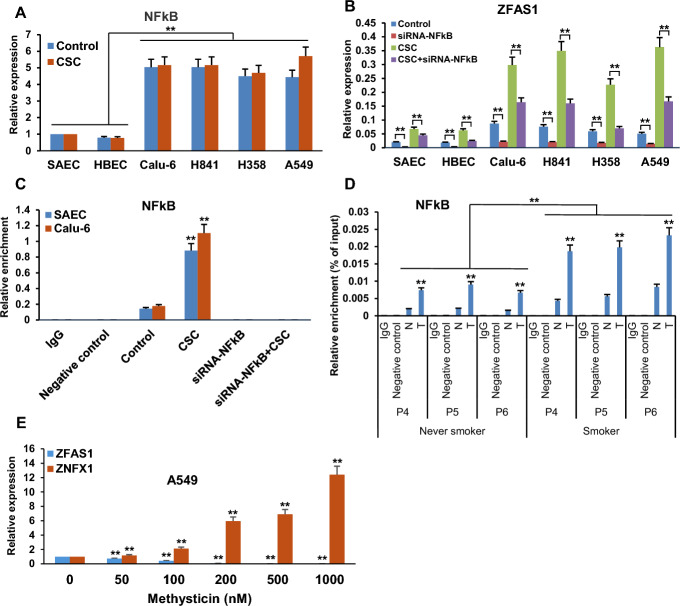
CSC upregulates *ZFAS1* via selective interaction of NFkB with the *ZFAS1* enhancer. **A** qRT-PCR analysis demonstrating expression of *NFkB-p65* in normal respiratory epithelia and lung cancer cells cultured in the presence or absence of CSC. **B** qRT-PCR analysis demonstrating that siRNA knockdown of *NFkB-p65* significantly decreased *ZFAS1* expression in normal respiratory epithelia and lung cancer cells treated with or without exposure to CSC. C/D) Quantitative ChIP analysis demonstrating increased levels of NFkB in the enhancer region of *ZFAS1* in SAEC and Calu-6 cells following CSC exposure (**C**) or in lung cancer tissues from both smokers and nonsmokers (**D**). **E** qRT-PCR analysis demonstrating *ZFAS1* and *ZNFX1* expression in A549 lung cancer cells treated with or without mythysticin at 0 to 1000 nM concentration for 48 h. Methysticin dose-dependently enhanced expression of *ZNFX1* while dose-dependently inhibiting expression of *ZFAS1* in normal respiratory epithelia and lung cancer cells. Data are mean ± SEM; T-test; *n* = 3; * *p* < 0.05; **p < 0.01

*CSC upregulates ZFAS1 via selective interaction of NFkB with the ZFAS1 enhancer.* Additional software-guided analysis identified five potential binding sites for NFkB within the 750-bp span of the *ZFAS1* enhancer (Fig. 9A). Since this master regulator has been implicated in tumor development and progression [44, 45], we next investigated if NFkB contributes to CSC-mediated activation of *ZFAS1*. qRT-PCR assays revealed that endogenous NFkB-p65 mRNA levels were higher in lung cancer cells relative to NREC; similar to what we observed for SP1, 5-day CSC exposure did not upregulate *NFkB-p65* expression (Fig. 11A). Knockdown of *NFkB-p65* repressed *ZFAS1*, and markedly attenuated CSC-induced upregulation of this gene  in NREC and lung cancer cells (Fig. 11B; Supplementary Fig. S11). CSC induced recruitment of NFkB-p65 to the enhancer region of *ZFAS1* in SAEC and Calu-6 cells (Fig. 11C). qChIP analysis demonstrated increased occupancy of NFkB-p65 in the *ZFAS1* enhancer region in primary lung cancers relative to paired normal lung tissues; NFkB-p65 occupancy within the regulatory region of *ZFAS1* was higher in cancer as well as normal lung tissues from smokers compared with never smokers (representative results depicted in Fig. 11D). Methysticin, a naturally occurring inhibitor of NFkB signaling [46], repressed *ZFAS1* while simultaneously upregulating *ZNFX1* in a dose-dependent manner in cultured A549 lung cancer cells (Fig. 11E).

## Discussion

Although the epidemiologic links between cigarette smoking and lung cancers are undeniable [47], the precise mechanisms by which tobacco smoke causes and advances these tumors remain unclear. In particular, the roles of noncoding RNAs in the pathogenesis of lung cancers have not been studied comprehensively. Our previous studies have demonstrated that cigarette smoke modulates microRNA expression in normal respiratory epithelia and lung cancer cells; CEBP/β-mediated upregulation of host gene for miR-31 and epigenetic repression of *miR-487b* activate Wnt signaling and upregulate *MYC*, *KRAS*, *EZH2*, and *SUZ12*, thereby enhancing a stem-like phenotype in pulmonary carcinomas [31, 33].

**Fig. 12:**
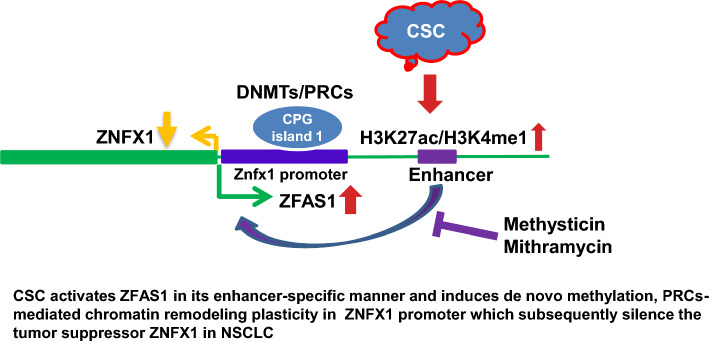
Mechanistic diagram summarizing mechanisms by which repression of *ZNFX1* by ZFAS1 mediates tobacco-induced pulmonary carcinogenesis. CSC activates *ZFAS1* in an enhancer-specific manner and induces de novo DNA methylation and polycomb-mediated chromatin remodeling within the *ZNFX1* promoter, which subsequently silences this tumor suppressor in NSCLC.

In the present study, we examined potential mechanisms by which perturbations of lncRNA expression contribute to pulmonary carcinogenesis (summarized in Fig. 12). Our analysis demonstrated that cigarette smoke modulates expression of numerous lncRNAs in NREC including ZFAS1, which has been implicated in diverse diseases including mycobacterial infections, epilepsy, rheumatoid arthritis, atherosclerosis, and cancer [48–50]. Activation of *ZFAS1* in cultured NREC and lung cancer lines by CSC coincided with the downregulation of *ZNFX1*. Consistent with these findings, *ZFAS1* expression was inversely correlated with *ZNFX1 *expression in primary lung tumor specimens, particularly those from smokers. Our experiments also demonstrated that lncRNA ZFAS1 binds to DNMT3A and DNMT3B, as well as EZH2, SUZ12, and BMI1. Knockdown or overexpression of *ZFAS1* significantly reduced or increased occupancy, respectively, of these epigenetic writers within the *ZNFX1* promoter. These findings suggest that lncRNA ZFAS1 functions as a scaffold to facilitate enrichment of epigenetic repressor proteins to the *ZNFX1* promoter in respiratory epithelial cells with cigarette smoke exposure, and are analogous to previous observations pertaining to recruitment of DNMTs and PRC-2-associated proteins to promoter targets by lncRNAs such as Dali, Dum, HOTAIR, and RepA [39, 40, 51–55]. Overexpression of *ZFAS1* enhanced growth of lung cancer cells in vitro and in vivo, suggesting that this lncRNA functions as an oncogene during pulmonary carcinogenesis. These latter findings confirm and extend observations by Zeng et al. [56 ]and Fan and colleagues [57] that ZFAS1 is elevated in NSCLC and enhances the growth and invasion of lung cancer cells by modulating the expression of *high mobility group AT-hook 2 (HMGA2)* and *fibroblast growth factor receptor substrate 2 (FRS2)*, respectively. Our findings are also consistent with previous observations that upregulation of *ZFAS1* is associated with the advanced stage of disease and decreased survival of patients with NSCLC [56, 58, 59]. Our experiments also demonstrated that overexpression of *ZNFX1* represses growth of lung tumor cells in vitro and in vivo; these observations suggest that *ZNFX1* encodes a novel tumor suppressor that is inactivated in lung cancers. As such, our studies are the first to implicate dysregulation of the *ZFAS1–ZNFX1* regulatory loop as a mechanism contributing to tobacco-induced pulmonary carcinogenesis.

Information available regarding the expression and function of ZNFX1 in normal and malignant cells is still highly limited. Encoded by an mRNA of relatively short half-life, this large protein (~ 2000AA) is a member of the helicase SF1 family that is involved in maintaining transgenerational RNAi-mediated gene silencing [60–62]. Additionally, *ZNFX1* has been identified as an interferon-stimulated gene (ISG), and ZNFX1 functions as a mitochondrial dsRNA sensor [63]. Mutations involving *ZNFX1* have been linked to several interferonopathy conditions including inherited susceptibility to mycobacterial infections [64–66]. In these clinical conditions, *ZNFX1* has been reported to be positively regulated by ZFAS1 [67], suggesting that the effects of *ZFAS1* upregulation on *ZNFX1* expression may be tissue and context specific. Aside from a single report that *ZNFX1* is upregulated in breast cancer cells following exposure to chemotherapeutic agents [68], the roles of *ZNFX1* during development and human carcinogenesis remain unknown.

Among normal tissues in the CCLE database (https://sites.broadinstitute.org/ccle/datasets), *ZNFX1* is highest in the lung and whole blood. Among cancer cell lines of diverse histologies in this database, ZNFX1 mRNA levels appear to be decreased in NSCLC and are considerably low in SCLC, which typically arise in individuals with extensive smoking histories and exhibit high mutational burden [4]. Although our studies suggest that *ZNFX1* is a tumor suppressor gene that is repressed in lung cancers, our analysis of the TCGA database identified no significant associations between ZNFX1 mRNA levels and overall survival of patients with NSCLC (data not shown). Presently, such analyses are not possible for SCLC patients. Additional studies are necessary to further delineate the roles of *ZNFX1* in normal cellular homeostasis as well as the mechanisms and clinical relevance of *ZNFX1* downregulation during pulmonary carcinogenesis. Furthermore, additional studies are necessary to fully delineate the targets and biological/clinical implications of *ZFAS1* activation in tobacco-induced lung cancers. Despite these limitations, our findings that SP1 and NFĸB inhibitors simultaneously repress *ZFAS1* and restore *ZNFX1* expression in lung cancer cells provide proof of concept that *ZFAS1–ZNFX1* dysregulation during pulmonary carcinogenesis is potentially druggable in the clinic.

## Supplementary Information

Supplementary material 1: S1. Summary of Affymetrix lncRNA (A) and mRNA (B) array analysis of CSC-mediated effects in cultured human SAEC. Differential expression of lncRNAs and mRNAs from Affymetrix array assays are shown in heatmaps. (C) Association analysis of consistently upregulated or downregulated lncRNAs and mRNAs following CSC exposure in SAEC cells was performed to select those paired lncRNAs and mRNAs that are located in the same chromosome and are less than 0.5 mb from each other. Thirteen paired lncRNAs and mRNAs are listed in this table. (D) Schematic depiction demonstrating the genomic relationship of *ZNFX1* and *ZFAS1*. (E) Fold changes of *ZNFX1* and *ZFAS1* induced by CSC in the array analysis.

Supplementary material 2: S2. Densitometry of Figs. 1E and 1F.

Supplementary material 3: S3. Correlation analyses of ZFAS1 and ZNFX1 in publicly available scRNA-seq datasets. (A) Cell population distribution of all epithelial cell types (AT1, AT2, Basal, Basal-d, Basal-px, Cil-px, Cilia, Club, Goblet, Ionocyte, Mucous, Proliferating epithelial, Serous) with cigarette smoking status from eight scRNA-seq cohorts (104 samples). (B) Correlation analyses of ZFAS1 and ZNFX1 in three epithelial subtypes (AT1, AT2, and Club). All three epithelial subtypes have more negative coefficients in smokers than never-smokers, with dramatic significance. The negative coefficients come from the exclusive expression of ZFAS1 and ZNFX1 in those epithelia. (C) Summarization of all 13 epithelial cell types with cell numbers, *p*-values, and correlation values in total, never-smoker, and smoker groups.

Supplementary material 4: S4. (A) qRT-PCR analysis of *ZFAS1 *expression in NREC and lung cancer cells exhibiting overexpression of *ZFAS1* relative to vector controls. (B) qRT-PCR analysis of *ZFAS1* expression in NREC and lung cancer cells before and after *ZFAS1* knockdown relative to vector controls. (C, D) Densitometry of Fig. 3B and D, respectively. *p < 0.05; **p < 0.01.

Supplementary material 5: S5. (A) qRT-PCR analysis demonstrating *ZNFX1* expression in NREC and lung cancer cells constitutively expressing *ZNFX1* relative to vector controls. (B) Immunoblot analysis demonstrating endogenous *ZNFX1* protein levels in Calu-6 and H841 cells with or without overexpression of *ZNFX1*. (C) qRT-PCR analysis demonstrating *ZNFX1* expression in NREC and lung cancer cells following knockdown of *ZNFX1* relative to vector controls. (D) Immunoblot analysis demonstrating endogenous ZNFX1 protein levels in SAEC and HBEC cells with or without depletion of ZNFX1. *p < 0.05; **p < 0.01.

Supplementary material 6: S6. (A) Schematic depiction demonstrating the promoter region of *ZNFX1* and putative enhancer region for *ZFAS1*. (B) Schematic distribution of 3 CpG islands in the first 2-kb promoter region of *ZNFX1*. (C) Schematic distribution of one CpG island in putative enhancer region for *ZFAS1* (40 kb upstream of *ZNFX1*). (D) MeDIP analysis of DNA methylation profiles in the second CpG island proximal to TSS of *ZNFX1* in SAEC and Calu-6 cells, demonstrating that CSC did not change DNA methylation in this region. (E) MeDIP analysis of DNA methylation in the CpG island within the regulatory element of *ZFAS1* in SAEC and Calu-6 cells; CSC did not alter DNA methylation in this region.

Supplementary material 7: S7. qRT-PCR analysis demonstrating that DAC increases *ZNFX1* expression in lung cancer cells, not normal lung epithelia. DAC abrogates *ZFAS1* overexpression-mediated repression of *ZNFX1* in normal lung epithelia and lung cancer cells.

Supplementary material 8: S8. MeDIP analysis of DNA methylation profiles in the first CpG island proximal to the TSS of *ZNFX1* in SAEC and Calu-6 cells; DAC decreases CSC- or *ZFAS1*-mediated DNA hypermethylation. *UBE2C* and *H19ICR* serve as negative and positive controls, respectively.

Supplementary material 9: S9. MSP analysis of DNA methylation profiles in the first CpG island proximal to the TSS of *ZNFX1* in human lung cancers relative to paired adjacent normal lung tissues (A) as well as in lung cancer cell lines compared with NREC (B). CpG methylation levels in the first CpG island proximal to TSS of *ZNFX1* (as evidenced by PCR products) were higher in tumors than corresponding normal lung tissues; DNA methylation in this region appeared to be higher in lung tumors from smokers relative to nonsmokers.

Supplementary material 10: S10. qRT-PCR analysis of *SP1* expression in NREC and lung cancer cells treated with or without siRNA targeting *SP1*.

Supplementary material 11: S11. qRT-PCR analysis of *NFkB-p65* expression in NREC and lung cancer cells treated with or without siRNA targeting *NFkB-p65*.

Supplementary material 12.

Supplementary material 13.

## Data Availability

The datasets used and/or analyzed during the current study are available from the corresponding author upon reasonable request.

## Abbreviations

CSC: Cigarette smoke condensate
NREC: Normal respiratory epithelial cells
LncRNA: Long noncoding RNAs
ZNFX1: Zinc finger NFX1-type containing 1
ZFAS1: ZNFX1 antisense 1
NSCLC: Non-small cell lung cancers
SCLC: Small cell lung cancers
PAH: Polycyclic aromatic hydrocarbons
MiRs: MicroRNAs
HOTAIR: HOX transcript antisense RNA
MALAT1: Metastasis-associated lung adenocarcinoma transcript 1
NEAT2: Nuclear-enriched abundant transcript 2
SCAL1: Smoking and cancer-associated lncRNA 1
HBEC: Human bronchial epithelial cells
SAEC: Small airway epithelial cells
MeDIP: Methylated DNA immunoprecipitation
MSP: Methylation specific PCR
qChIP: Quantitative chromatin immunoprecipitation
CLIP: Cross-link immunoprecipitation
FAIRE: Formaldehyde-assisted isolation of regulatory elements
SP1: Specificity Protein 1
m5C: 5-Methylcytidine
RIP: RNA immunoprecipitation
