# Supplementary material for: Repression of ZNFX1 by LncRNA ZFAS1 mediates tobacco-induced pulmonary carcinogenesis
Source: Cell Mol Biol Lett. 2025 Apr 10;30:44. doi: 10.1186/s11658-025-00705-x (PMC11983736; doi:10.1186/s11658-025-00705-x)

A

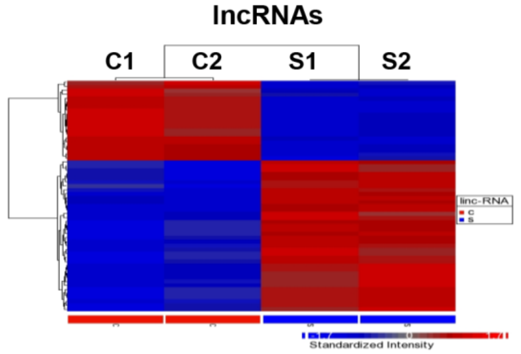

B

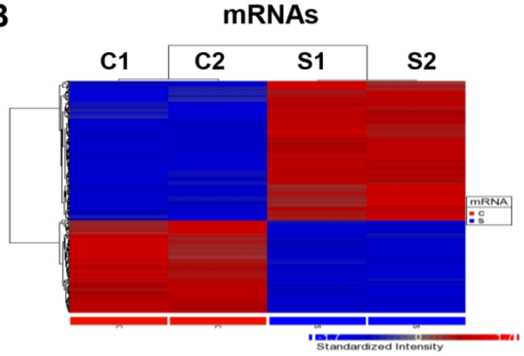

C

Association analysis of lncRNAs and their adjacent genes

lncRNA	Gene ID	Gene name	lncRNA Gene name	Fold change in mRNA	Fold change in lncRNA	Chromosome	Strand	Distance
ASLNC16067	NM_003768	PEA15	AJ844623	-2.0383	-2.000058	chr1	+	279852
ASLNC24236	NM_003755	EIF3S4	BX648637	1.69563	-4.677296	chr19	-	165884
ASLNC09164	NM_006848	CCDC85B	uc001odz	3.498304	-3.254462	chr11	+	386795
ASLNC23806	NM_144615	TMIGD2	BC073825	16.46955	-1.475106	chr19	-	57939
ASLNC08994	NM_001997	FAU	uc001ody	1.599833	-3.716542	chr11	-	377298
ASLNC09008	NM_006848	CCDC85B	uc001odv	3.498304	-2.566315	chr11	+	465642
ASLNC02713	NM_006848	CCDC85B	HIV1664	3.498304	-3.099634	chr11	+	463902
ASLNC09527	NM_001017958	OS9	uc009zpz	1.209381	9.678603	chr12	+	67952
ASLNC07968	NM_001039708	SDCCAG3	uc004cip	2.740683	5.17019	chr9	-	314054
ASLNC16690	NM_021035	ZNFX1	ZFAS1	-3.591133	2.409348	chr20	-	<200
ASLNC14608	NM_023936	MRPS34	BC011661	2.043304	2.257819	chr16	-	188921
ASLNC09278	NM_016564	CEND1	uc001lsq	9.031796	1.416929	chr11	-	90740
ASLNC09286	NM_016564	CEND1	uc001lsr	9.031796	1.604605	chr11	-	91349

D

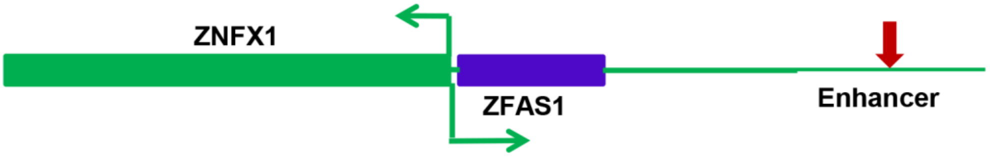

E

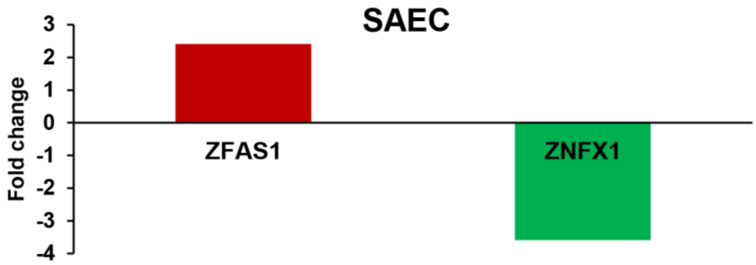

Supplement: Supplementary file 1 — Supplementary material 1: S1. Summary of Affymetrix lncRNA (A) and mRNA (B) array analysis of CSC-mediated effects in cultured human SAEC. Differential expression of lncRNAs and mRNAs from Affymetrix array assays are shown in heatmaps. (C) Association analysis of consistently upregulated or downregulated lncRNAs and mRNAs following CSC exposure in SAEC cells was performed to select those paired lncRNAs and mRNAs that are located in the same chromosome and are less than 0.5 mb from each other. Thirteen paired lncRNAs and mRNAs are listed in this table. (D) Schematic depiction demonstrating the genomic relationship of ZNFX1 and ZFAS1. (E) Fold changes of ZNFX1 and ZFAS1 induced by CSC in the array analysis. [file 11658_2025_705_MOESM1_ESM.pdf]
