# Supplementary figures and images for: Repression of ZNFX1 by LncRNA ZFAS1 mediates tobacco-induced pulmonary carcinogenesis
Source: Cell Mol Biol Lett. 2025 Apr 10;30:44. doi: 10.1186/s11658-025-00705-x (PMC11983736; doi:10.1186/s11658-025-00705-x)

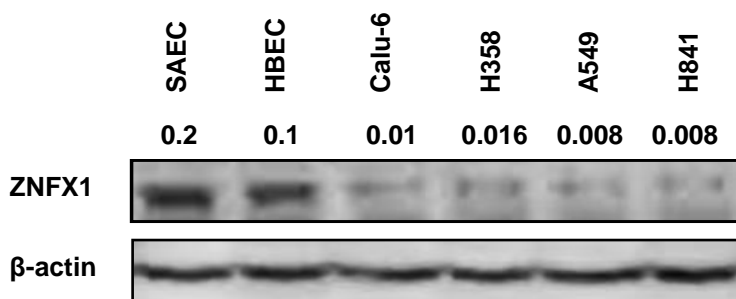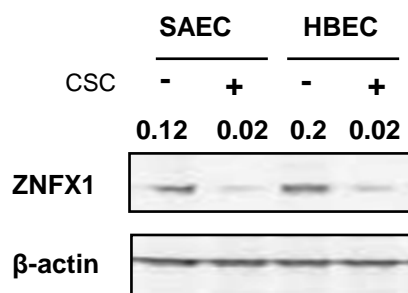

Supplement: Supplementary file 2 — Supplementary material 2: S2. Densitometry of Figs. 1E and 1F. [file 11658_2025_705_MOESM2_ESM.pdf]

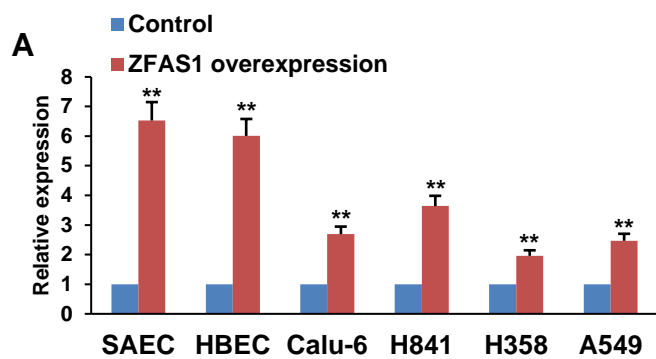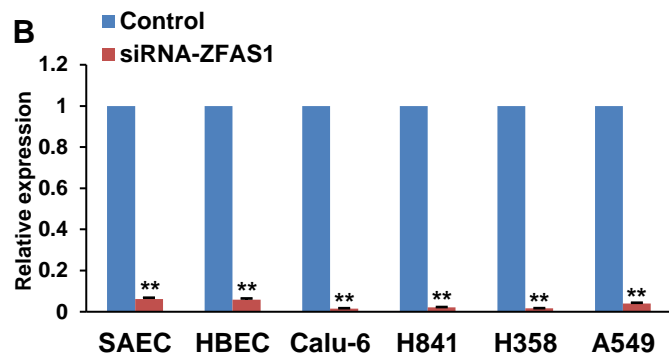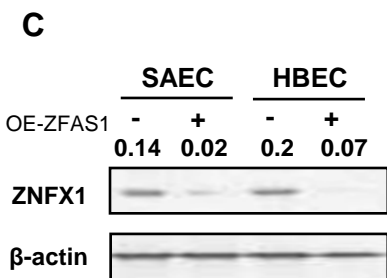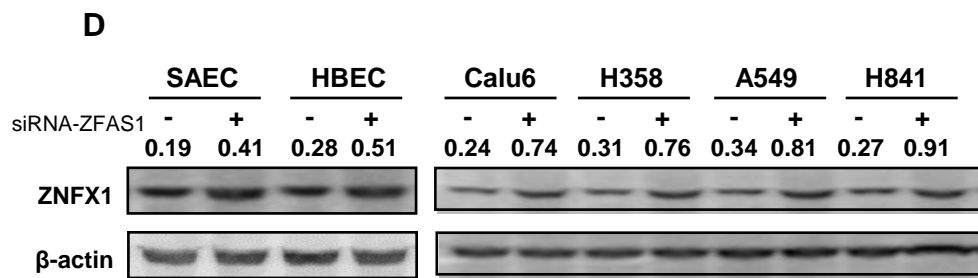

Supplement: Supplementary file 4 — Supplementary material 4: S4. (A) qRT-PCR analysis of ZFAS1 expression in NREC and lung cancer cells exhibiting overexpression of ZFAS1 relative to vector controls. (B) qRT-PCR analysis of ZFAS1 expression in NREC and lung cancer cells before and after ZFAS1 knockdown relative to vector controls. (C, D) Densitometry of Fig. 3B and D, respectively. *p < 0.05; **p < 0.01. [file 11658_2025_705_MOESM4_ESM.pdf]

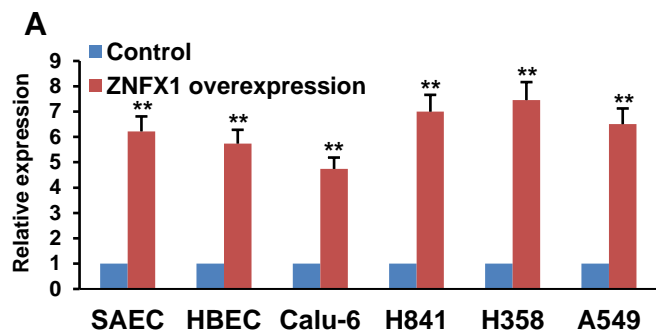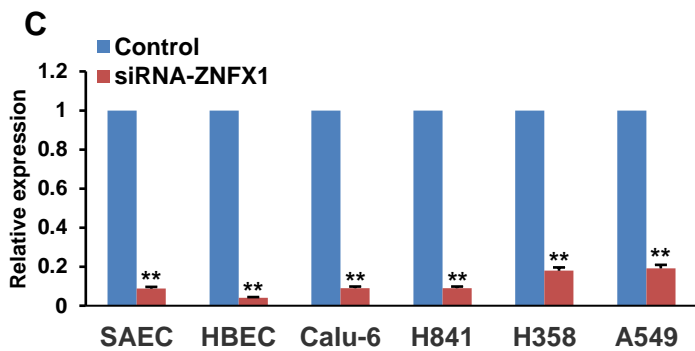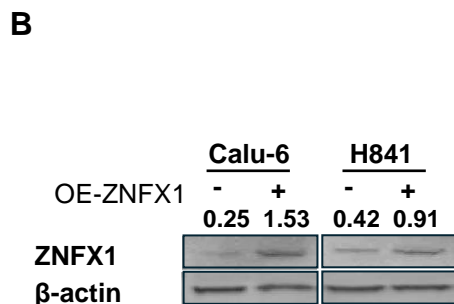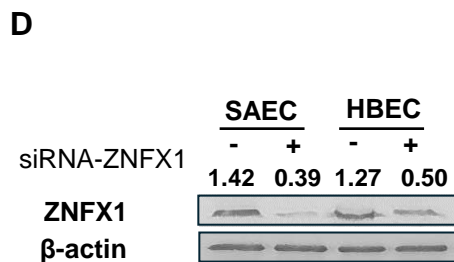

Supplement: Supplementary file 5 — Supplementary material 5: S5. (A) qRT-PCR analysis demonstrating ZNFX1 expression in NREC and lung cancer cells constitutively expressing ZNFX1 relative to vector controls. (B) Immunoblot analysis demonstrating endogenous ZNFX1 protein levels in Calu-6 and H841 cells with or without overexpression of ZNFX1. (C) qRT-PCR analysis demonstrating ZNFX1 expression in NREC and lung cancer cells following knockdown of ZNFX1 relative to vector controls. (D) Immunoblot analysis demonstrating endogenous ZNFX1 protein levels in SAEC and HBEC cells with or without depletion of ZNFX1. *p < 0.05; **p < 0.01. [file 11658_2025_705_MOESM5_ESM.pdf]

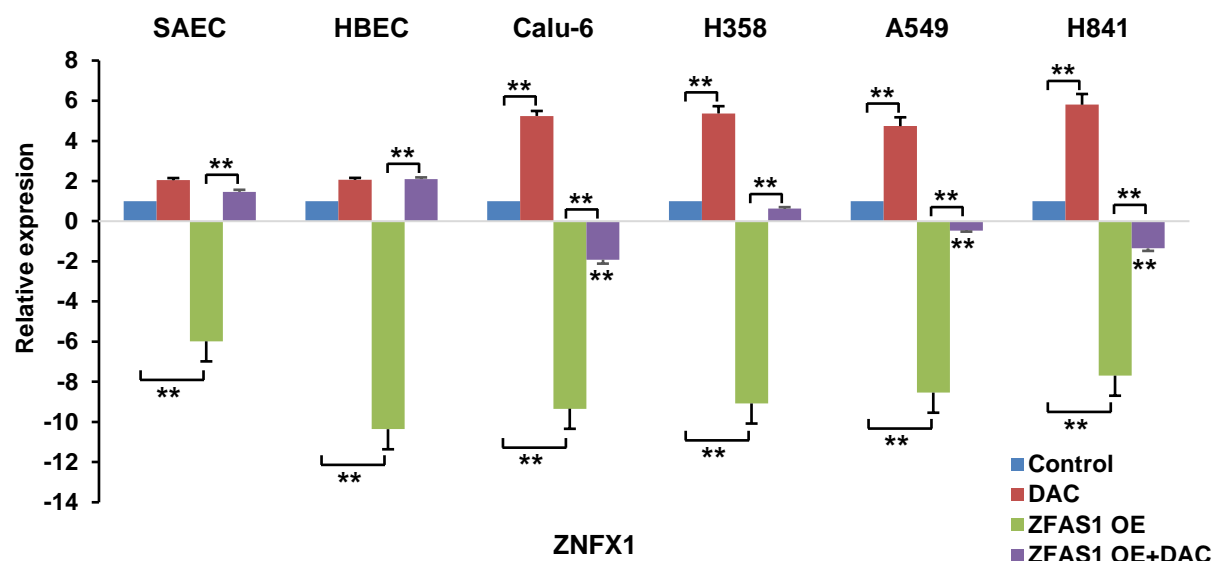

Supplement: Supplementary file 7 — Supplementary material 7: S7. qRT-PCR analysis demonstrating that DAC increases ZNFX1 expression in lung cancer cells, not normal lung epithelia. DAC abrogates ZFAS1 overexpression-mediated repression of ZNFX1 in normal lung epithelia and lung cancer cells. [file 11658_2025_705_MOESM7_ESM.pdf]

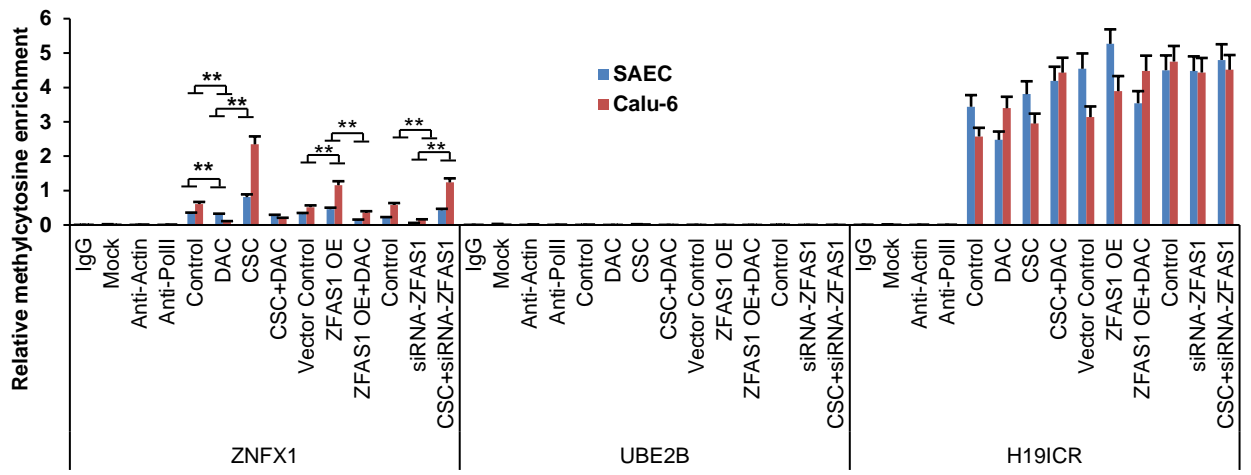

Supplement: Supplementary file 8 — Supplementary material 8: S8. MeDIP analysis of DNA methylation profiles in the first CpG island proximal to the TSS of ZNFX1 in SAEC and Calu-6 cells; DAC decreases CSC- or ZFAS1-mediated DNA hypermethylation. UBE2C and H19ICR serve as negative and positive controls, respectively. [file 11658_2025_705_MOESM8_ESM.pdf]

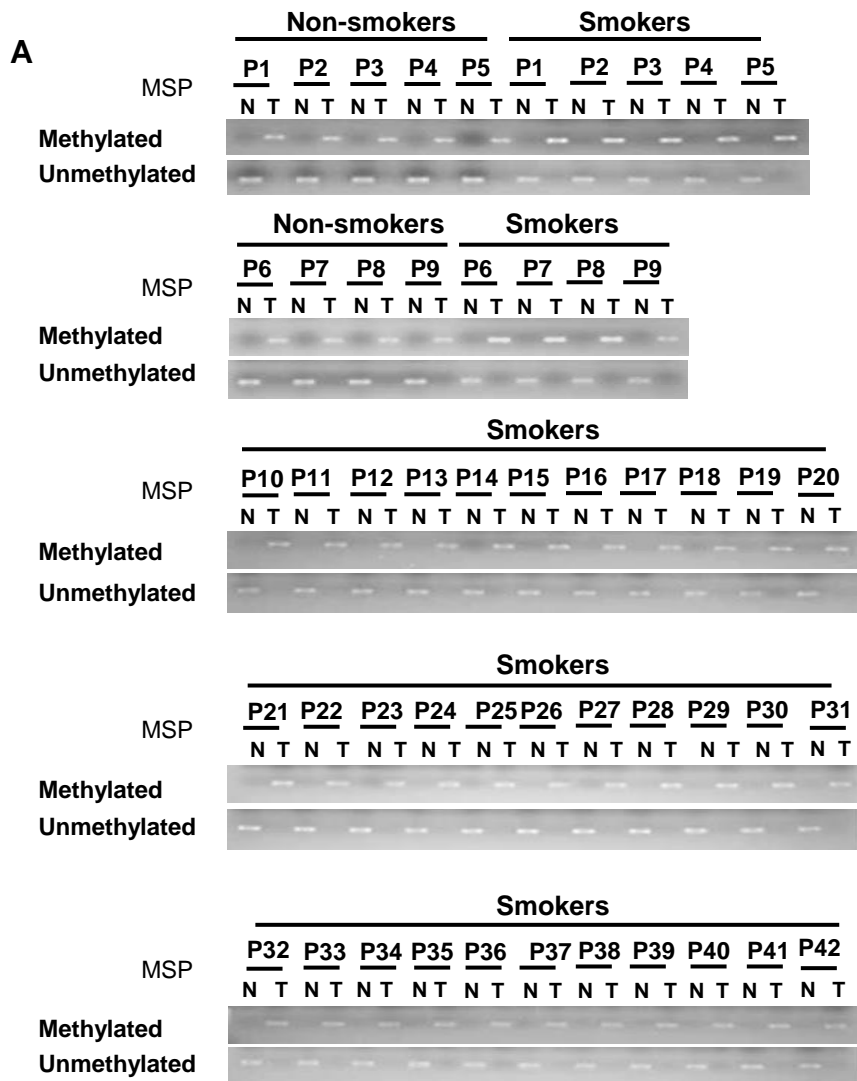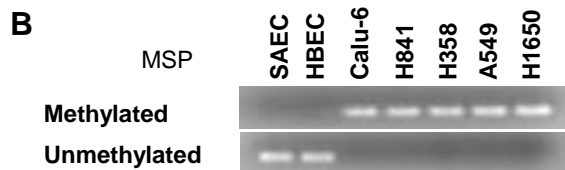

Supplement: Supplementary file 9 — Supplementary material 9: S9. MSP analysis of DNA methylation profiles in the first CpG island proximal to the TSS of ZNFX1 in human lung cancers relative to paired adjacent normal lung tissues (A) as well as in lung cancer cell lines compared with NREC (B). CpG methylation levels in the first CpG island proximal to TSS of ZNFX1 (as evidenced by PCR products) were higher in tumors than corresponding normal lung tissues; DNA methylation in this region appeared to be higher in lung tumors from smokers relative to nonsmokers. [file 11658_2025_705_MOESM9_ESM.pdf]

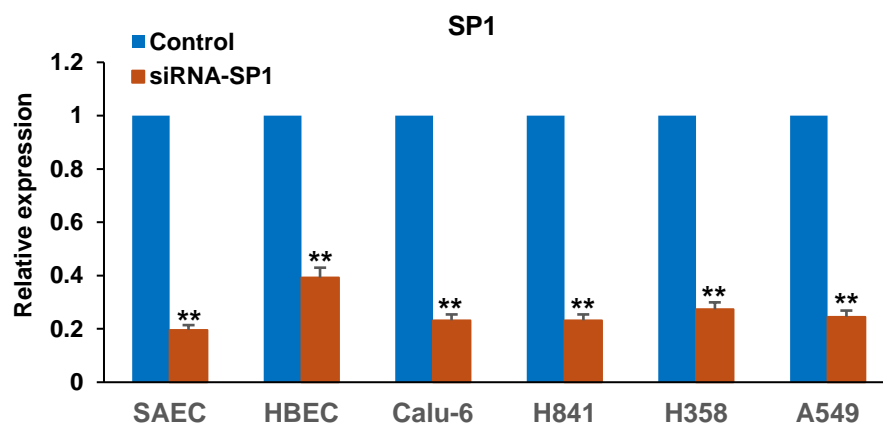

Supplement: Supplementary file 10 — Supplementary material 10: S10. qRT-PCR analysis of SP1 expression in NREC and lung cancer cells treated with or without siRNA targeting SP1. [file 11658_2025_705_MOESM10_ESM.pdf]

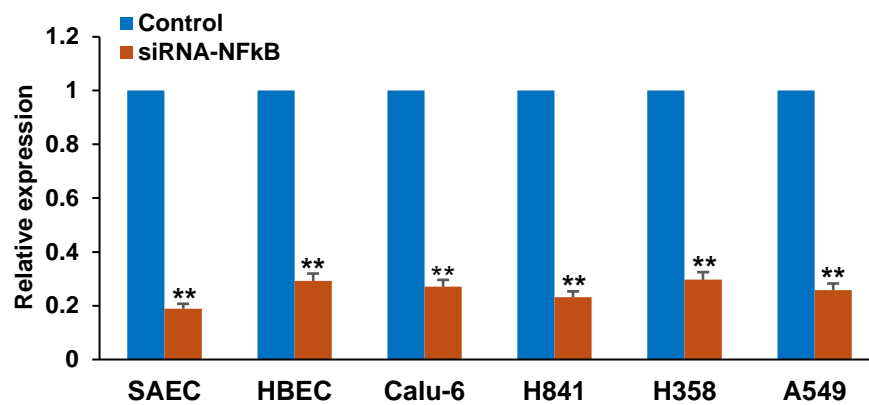

\*\*

Supplement: Supplementary file 11 — Supplementary material 11: S11. qRT-PCR analysis of NFkB-p65 expression in NREC and lung cancer cells treated with or without siRNA targeting NFkB-p65. [file 11658_2025_705_MOESM11_ESM.pdf]
