# Supplementary material for: Repression of ZNFX1 by LncRNA ZFAS1 mediates tobacco-induced pulmonary carcinogenesis
Source: Cell Mol Biol Lett. 2025 Apr 10;30:44. doi: 10.1186/s11658-025-00705-x (PMC11983736; doi:10.1186/s11658-025-00705-x)
Supplement: Supplementary file 3 — Supplementary material 3: S3. Correlation analyses of ZFAS1 and ZNFX1 in publicly available scRNA-seq datasets. (A) Cell population distribution of all epithelial cell types (AT1, AT2, Basal, Basal-d, Basal-px, Cil-px, Cilia, Club, Goblet, Ionocyte, Mucous, Proliferating epithelial, Serous) with cigarette smoking status from eight scRNA-seq cohorts (104 samples). (B) Correlation analyses of ZFAS1 and ZNFX1 in three epithelial subtypes (AT1, AT2, and Club). All three epithelial subtypes have more negative coefficients in smokers than never-smokers, with dramatic significance. The negative coefficients come from the exclusive expression of ZFAS1 and ZNFX1 in those epithelia. (C) Summarization of all 13 epithelial cell types with cell numbers, p-values, and correlation values in total, never-smoker, and smoker groups. [file 11658_2025_705_MOESM3_ESM.pdf]

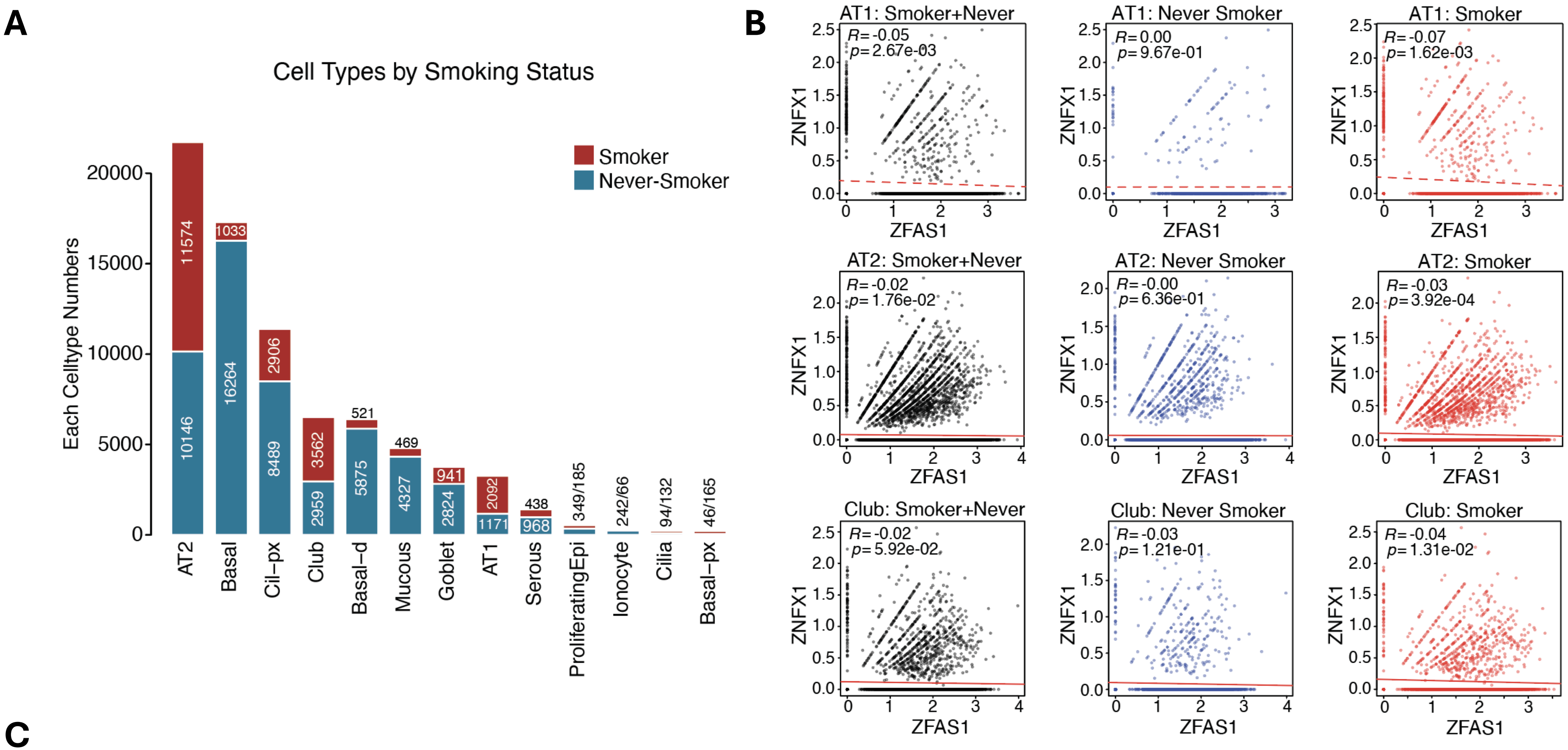

C

Cell_Type	ALL Number of Cell	All_Cor	All_Pvalue	Never Number of Cell	Never_Cor	Never_Pvalue	Smoker Number of Cell	Smoker_Cor	Smoker_Pvalue
All_cells	77838	-0.0633598	4.62E-70	53754	-0.065385489	5.14E-52	24084	-0.081659881	6.44E-37
**AT1**	**3263**	**-0.0525712**	**0.0026651**	**1171**	**0.001221192**	**0.966702439**	**2092**	**-0.068889628**	**0.00161733**
**AT2**	**21720**	**-0.016101**	**0.0176473**	**10146**	**-0.004699599**	**0.635983166**	**11574**	**-0.03295219**	**0.000391632**
Serous	1406	0.10353134	0.0001006	968	0.040317623	0.210104244	438	0.097802443	0.040765086
**Club**	**6521**	**-0.0239752**	**0.0528714**	**2959**	**-0.028535193**	**0.120690882**	**3562**	**-0.041579979**	**0.013072097**
Proliferating Epithel	534	-0.0979938	0.0235354	349	-0.122051469	0.02258033	185	-0.111748643	0.129926647
Cil-px	11395	0.03322843	0.0003887	8489	0.014710734	0.175335203	2906	0.042440455	0.022143461
Basal-px	211	-0.0514622	0.4571281	46	-0.123910115	0.411965437	165	0.074821005	0.33951384
Mucous	4796	0.01485967	0.3035417	4327	0.039080788	0.010141379	469	-0.064834063	0.160974595
Basal	17297	0.00588832	0.438711	16264	0.01593968	0.04207612	1033	0.026806474	0.389414253
Goblet	3765	0.01841603	0.2585938	2824	0.012874097	0.494056002	941	-0.025626042	0.432347201
Basal-d	6396	-0.0017491	0.8887758	5875	0.003526335	0.786981948	521	0.004480461	0.91873819
Ionocyte	308	0.03696789	0.5180418	242	0.039389996	0.541975346	66	0.053082688	0.672072892
Cilia	226	-0.0682031	0.3073394	94	-0.191111006	0.065016542	132	0.032563145	0.710889276
