# Supplementary material for: Repression of ZNFX1 by LncRNA ZFAS1 mediates tobacco-induced pulmonary carcinogenesis
Source: Cell Mol Biol Lett. 2025 Apr 10;30:44. doi: 10.1186/s11658-025-00705-x (PMC11983736; doi:10.1186/s11658-025-00705-x)
Supplement: Supplementary file 6 — Supplementary material 6: S6. (A) Schematic depiction demonstrating the promoter region of ZNFX1 and putative enhancer region for ZFAS1. (B) Schematic distribution of 3 CpG islands in the first 2-kb promoter region of ZNFX1. (C) Schematic distribution of one CpG island in putative enhancer region for ZFAS1 (40 kb upstream of ZNFX1). (D) MeDIP analysis of DNA methylation profiles in the second CpG island proximal to TSS of ZNFX1 in SAEC and Calu-6 cells, demonstrating that CSC did not change DNA methylation in this region. (E) MeDIP analysis of DNA methylation in the CpG island within the regulatory element of ZFAS1 in SAEC and Calu-6 cells; CSC did not alter DNA methylation in this region. [file 11658_2025_705_MOESM6_ESM.pdf]

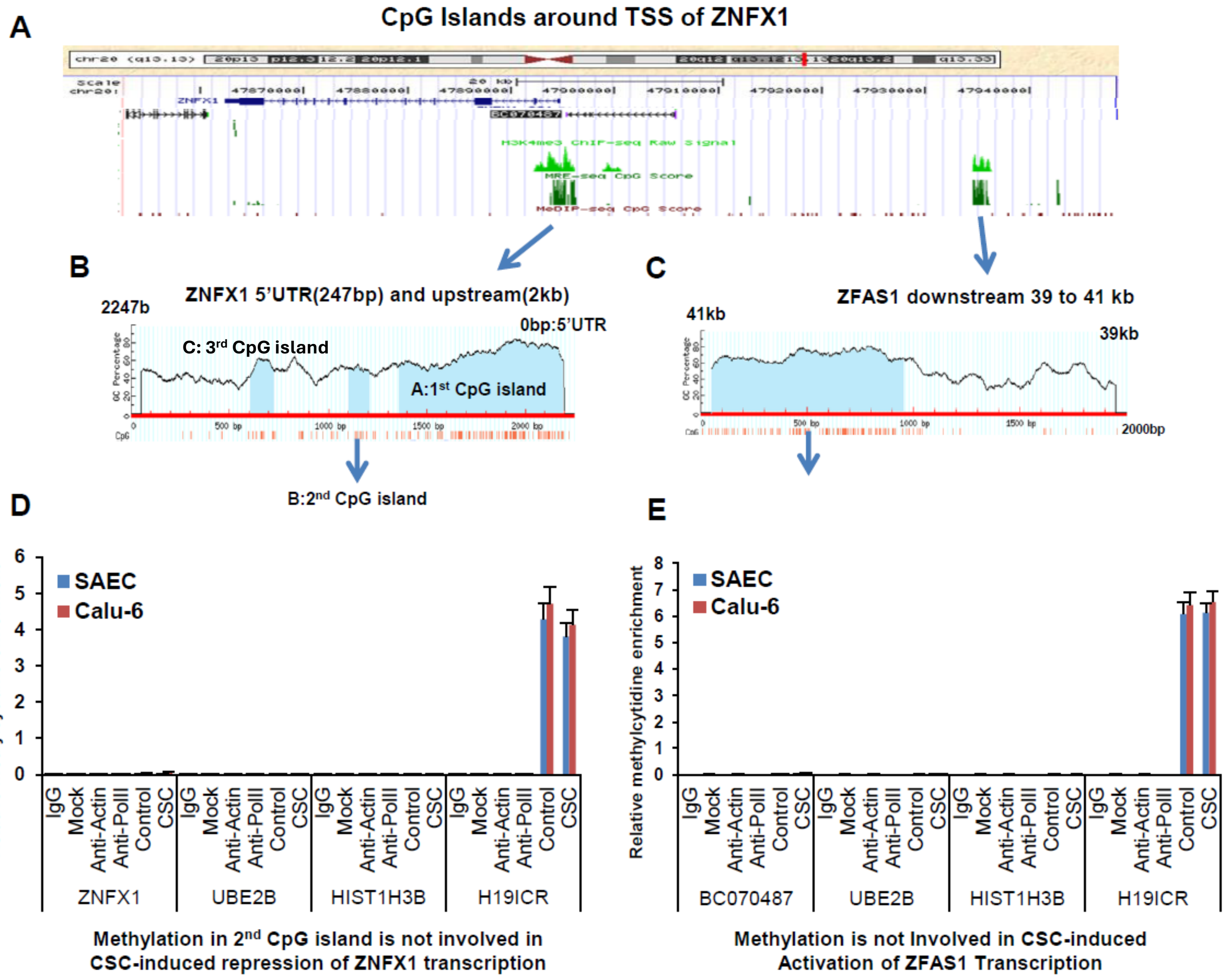

A
CpG Islands around TSS of ZNFX1
B
ZNFX1 5'UTR(247bp) and upstream(2kb)
2247b
0bp:5'UTR
C: 3rd CpG island
A:1st CpG island
B:2nd CpG island
C
ZFAS1 downstream 39 to 41 kb
41kb
39kb
2000bp
D
SAEC
Calu-6
Relative methylcytidine enrichment
IgG
Mock
Anti-Actin
Anti-PolII
Control
CSC
ZNFX1
UBE2B
HIST1H3B
H19ICR
Methylation in 2nd CpG island is not involved in CSC-induced repression of ZNFX1 transcription
E
SAEC
Calu-6
Relative methylcytidine enrichment
BC070487
UBE2B
HIST1H3B
H19ICR
Methylation is not Involved in CSC-induced Activation of ZFAS1 Transcription
