# Supplementary material for: Repression of ZNFX1 by LncRNA ZFAS1 mediates tobacco-induced pulmonary carcinogenesis
Source: Cell Mol Biol Lett. 2025 Apr 10;30:44. doi: 10.1186/s11658-025-00705-x (PMC11983736; doi:10.1186/s11658-025-00705-x)
Supplement: Supplementary file 12 — Supplementary material 12. [file 11658_2025_705_MOESM12_ESM.docx]

**Supplementary Table 1: Primer sequences**

**ChIP primers**

| Name |  | Sequence |
| --- | --- | --- |
| ZNFX1-P-F |  | 5’-AAG AAC GAA TGG CTT CCT GA-3’ |
| ZNFX1-P-R |  | 5’-CGG CCT GGA CAA CTA CTA GA-3’ |
|  |  |  |
|  |  |  |
|  |  |  |
|  |  |  |
|  |  |  |
|  |  |  |
| ZFAS1-enhancer-P-F |  | 5’-GGA GGC AAT GAG GAG CTC TG -3’ |
| ZFAS1-enhancer-P-R |  | 5’-TCT TGT GTA GCC TGC CTT GAG -3’ |
|  |  |  |
|  |  |  |
|  |  |  |
|  |  |  |

**MEDIP primers**

| Name |  | Sequence |
| --- | --- | --- |
| ZNFX1-1^st^ CpG island-F |  | 5’-CAA AGA AGG GAA GGG AGG AC-3’ |
| ZNFX1-1^st^ CpG island-R |  | 5’-GCG AAA GCC ATC TTT GGT TA-3’ |
|  |  |  |
|  |  |  |
| ZNFX1-2nd CpG island-F |  | 5’-TGG CTG CTC TAA CCT GTG AA-3’ |
| ZNFX1-2nd CpG island-R |  | 5’- CCC ATC AGA TCG ACA ATG C -3’ |
|  |  |  |
|  |  |  |
| H19 ICR-F |  | 5'-GAG CCG CAC CAG ATC TTC AG-3' |
| H19 ICR-R |  | 5'-TTG GTG GAA CAC ACT GTG ATC A-3' |
| UBE2B-F |  | 5'-CTC AGG GGT GGA TTG TTG AC-3' |
| UBE2B-R |  | 5'-TGT GGA TTC AAA GAC CAC GA-3' |
| H3B-F |  | 5'-CCC ACA CTT CTT ATG CGA CA-3' |
| H3B-R |  | 5'-CTG TGC CTG GTT GCA GAT TA-3' |

**CLIP primers**

| Name |  | Sequence |
| --- | --- | --- |
|  |  |  |
|  |  |  |
|  |  |  |
|  |  |  |
| ZAFS1-CLIP-F |  | 5'-GCG AAA GCC ATC TTT GGT TA-3' |
| ZFAS1-CLIP-R |  | 5'-CGT ATG AAG CCT GAC TGC AA-3' |

**FAIRE primers**

| Name |  | Sequence |  |
| --- | --- | --- | --- |
| ZFAS1-RE-F1 |  | 5'-CAC ATA GAG CCT GGT TCC CTG-3' | |
| AFAS1-RE-R1 |  | 5'-CTA CCG TCC AAA CTT TGC GC-3' | |
| ZFAS1-RE-F2 |  | 5'-GTA TCG CCC CTG TCT TCC TG-3' | |
| ZFAS1-RE-R2 |  | 5'-TGT AGC CTG CCT TGA GTT GG-3' | |
| ZFAS1-RE-F3 |  | 5'-AAG AGC TGC GCA AAG TTT GG-3' | |
| ZFAS1-RE-R3 |  | 5'-GAG CGA CAG GGA CAG GAT TC-3' | |
| ZFAS1-RE-F4 |  | 5'-GAA TCC TGT CCC TGT CGC TC-3' | |
| ZFAS1-RE-R4 |  | 5'-GGC AGA CCC GCG TAT AGT C-3' | |
|  |  |  | |
|  |  |  | |
|  |  |  | |
|  |  |  | |
|  |  |  | |
|  |  |  | |
|  |  |  | |
|  |  |  | |
| ZFAS1-U-F1 |  | 5'-TGT GGG TTT GGT GGG TTT GT-3' | |
| ZFAS1-U-R1 |  | 5'-AAA CCT GAG GCT CTT GGG TG-3' | |
| ZFAS1-U-F2 |  | 5'-ACA CAT AGA GCC TGG TTC CC-3' | |
| ZFAS1-U-R2 |  | 5'-CTA CCG TCC AAA CTT TGC GC-3' | |
| ZFAS1-U-F3 |  | 5'-GTA TCG CCC CTG TCT TCC TG-3' | |
| ZFAS1-U-R3 |  | 5'-CTG TCA CCG AGC TGA TGT CC-3' | |
| ZFAS1-D-F1 |  | 5'-TGC TCA CAG GAC TTG AAG GG-3' | |
| ZFAS1-D-R1 |  | 5'-CTC CAA GCT TCA CGA CCC C-3' | |
| ZFAS1-D-F2 |  | 5'-GGG AAA TGA GCA CAA GAG GC-3' | |
| ZFAS1-D-R2 |  | 5'-AAA TCT GGC GCC CTA CCT C-3' | |

**Bisulfite primers**

| Name |  | Sequence |
| --- | --- | --- |
| ZNFX1-1^st^ CpG island-F-1 |  | 5’-GTA TAA AGA AGG GAA GGG AGG ATT A-3’ |
| ZNFX1-1^st^ CpG island-R-1 |  | 5’-TAA AAA AAA TTC AAA AAA CCA TTC-3’ |
| ZNFX1-1^st^ CpG island-F-2 |  | 5’-GTG TTT TTT ATT TTG GGT TTT-3’ |
| ZNFX1-1^st^ CpG island-R-2 |  | 5’-ATC TAA ATC ACC TAA ATT CCT TCA C-3’ |
|  |  |  |
|  |  |  |
|  |  |  |
|  |  |  |

**MSP primers**

| Name |  | Sequence |
| --- | --- | --- |
|  |  |  |
|  |  |  |
| ZNFX1-1^st^ CpG island-MSP-M-F |  | 5’-GAA GGG AGG ATT AGA GGT TTT TAA C-3’ |
| ZNFX1-1^st^ CpG island-MSP-M-R |  | 5’-CCT AAT TAC AAT CAA ACT TCA TAC GC-3’ |
| ZNFX1-1^st^ CpG island-MSP-U-F |  | 5’-GAA GGG AGG ATT AGA GGT TTT TAA T -3’ |
| ZNFX1-1^st^ CpG island-MSP-U-R |  | 5’-CTA ATT ACA ATC AAA CTT CAT ACA CT-3’ |

**Expression primers/ siRNAs**

| **Name** | **Vendor** | **Location** | **Catalogue #** |
| --- | --- | --- | --- |
|  |  |  |  |
| ZNFX1 | Thermo Fisher | Grand Island, NY | Hs01105231-m1 |
| ZFAS1 | Thermo Fisher | Grand Island, NY | Hs01379985-m1 |
| SP1 | Thermo Fisher | Grand Island, NY | Hs00916521-m1 |
| P65/RELA | Thermo Fisher | Grand Island, NY | Hs01042014 |
| siRNA-ZNFX1 | Thermo Fisher | Grand Island, NY | ID:125311 |
| siRNA-ZAFS1 | Thermo Fisher | Grand Island, NY | ID:271502 |
| siRNA-SP1 | Thermo Fisher | Grand Island, NY | ID:116546 |
| siRNA-P65/RELA | Cell Signaling | Danvers, MA | 6261S |
| GAPDH | Thermo Fisher | Grand Island, NY | Hs0442032­_g1 |
| GAPDH | Origene | Rockville, MD | HP205798 |
| β-actin | Thermo Fisher/ABI | Grand Island, NY | LOT 1407034 |

|  |  |  |
| --- | --- | --- |
| \|  \|  \|  \|  \| \| --- \| --- \| --- \| --- \| \|  \|  \|  \|  \| \|  \|  \|  \|  \| \|  \|  \|  \|  \| \|  \|  \|  \|  \| \|  \|  \|  \|  \| \|  \|  \|  \|  \| \|  \|  \|  \|  \| \|  \|  \|  \|  \| \|  \|  \|  \|  \| \|  \|  \|  \|  \| |  |  |
|  |  |  |
|  |  |  |
|  |  |  |
|  |  |  |
|  |  |  |
|  |  |  |
|  |  |  |
|  |  |  |
|  |  |  |
|  |  |  |
|  |  |  |
