# Supplementary material for: Repression of ZNFX1 by LncRNA ZFAS1 mediates tobacco-induced pulmonary carcinogenesis
Source: Cell Mol Biol Lett. 2025 Apr 10;30:44. doi: 10.1186/s11658-025-00705-x (PMC11983736; doi:10.1186/s11658-025-00705-x)
Supplement: Supplementary file 13 — Supplementary material 13. [file 11658_2025_705_MOESM13_ESM.docx]

**Supplementary Table 2: Antibodies**

| **Name** | **Vendor** | **Location** | **Catalogue #** |
| --- | --- | --- | --- |
|  |  |  |  |
| ZNFX1 | Abcam | Cambridge, MA | Ab179452 |
| SP1 | Thermo Fisher | Grand Island, NY | PA5-29165 |
| H3K4me1 | Thermo Fisher | Carlsbad, CA | 710795 |
| H3K4me3 | Millipore | Billerica, MA | 04-085 |
| H3K27ac | Thermo Fisher | Carlsbad, CA | MA5-23516 |
| H3K27me3 | Millipore | Billerica, MA | sc-32877 |
| RNA polymerase II | Millipore | Billerica, MA | 05-623B |
| EZH2 | Abcam | Cambridge, MA | ab307646 |
| SUZ12 | Abcam | Cambridge, MA | ab12073 |
| BMI1 | Abcam | Cambridge, MA | ab254253 |
| DNMT1 | Thermo Fisher | Grand Island, NY | MA5-16169 |
| DNMT3A | Thermo Fisher | Grand Island, NY | PA1-882 |
| DNMT3B | Thermo Fisher | Grand Island, NY | MA5-51459 |
| 5-methylcytosine | Abcam | Cambridge, MA | ab10805 |
| P65/RELA | Abcam | Cambridge, MA | ab32536 |
| P65 (acetyl K310) | Abcam | Cambridge, MA | ab218533 |
| GAPDH | Cell Signaling | Danvers, MA | 2118S |
| β-actin | Santa Cruz Biotechnology, Inc. | Santa Cruz, CA | sc-1616 |
|  |  |  |  |
|  |  |  |  |
